# Comparison of the Mitochondrial Genomes and Steady State Transcriptomes of Two Strains of the Trypanosomatid Parasite, *Leishmania tarentolae*

**DOI:** 10.1371/journal.pntd.0003841

**Published:** 2015-07-23

**Authors:** Larry Simpson, Stephen M. Douglass, James A. Lake, Matteo Pellegrini, Feng Li

**Affiliations:** 1 Department of Microbiology, Immunology and Molecular Genetics, Geffen School of Medicine at UCLA, University of California, Los Angeles, Los Angeles, California, United States of America; 2 Bioinformatics Interdepartmental Program, University of California, Los Angeles, Los Angeles, California, United States of America; 3 Department of Molecular, Cellular and Developmental Biology, University of California, Los Angeles, Los Angeles, California, United States of America; 4 Dental Research Institute, School of Dentistry, University of California, Los Angeles, Los Angeles, California, United States of America; Seattle Biomedical Research Institute, UNITED STATES

## Abstract

U-insertion/deletion RNA editing is a post-transcriptional mitochondrial RNA modification phenomenon required for viability of trypanosomatid parasites. Small guide RNAs encoded mainly by the thousands of catenated minicircles contain the information for this editing. We analyzed by NGS technology the mitochondrial genomes and transcriptomes of two strains, the old lab UC strain and the recently isolated LEM125 strain. PacBio sequencing provided complete minicircle sequences which avoided the assembly problem of short reads caused by the conserved regions. Minicircles were identified by a characteristic size, the presence of three short conserved sequences, a region of inherently bent DNA and the presence of single gRNA genes at a fairly defined location. The LEM125 strain contained over 114 minicircles encoding different gRNAs and the UC strain only ~24 minicircles. Some LEM125 minicircles contained no identifiable gRNAs. Approximate copy numbers of the different minicircle classes in the network were determined by the number of PacBio CCS reads that assembled to each class. Mitochondrial RNA libraries from both strains were mapped against the minicircle and maxicircle sequences. Small RNA reads mapped to the putative gRNA genes but also to multiple regions outside the genes on both strands and large RNA reads mapped in many cases over almost the entire minicircle on both strands. These data suggest that minicircle transcription is complete and bidirectional, with 3’ processing yielding the mature gRNAs. Steady state RNAs in varying abundances are derived from all maxicircle genes, including portions of the repetitive divergent region. The relative extents of editing in both strains correlated with the presence of a cascade of cognate gRNAs. These data should provide the foundation for a deeper understanding of this dynamic genetic system as well as the evolutionary variation of editing in different strains.

## Author Summary

U-insertion/deletion RNA editing is a unique post-transcriptional mRNA modification process that occurs in trypanosomatid parasites and is required for viability. The participation of guide RNAs which are transcribed from the thousands of catenated minicircles in determining the precise sites and number of U’s inserted and deleted to create translatable mRNAs is novel and significant in terms of the recently realized importance of small RNAs in biology. This study contributes the necessary bioinformatics foundation for a deeper understanding of this important genetic system in molecular detail using a model trypanosomatid, *Leishmania tarentolae*. We used Next Generation Sequencing methods to determine the complete maxicircle and minicircle genomes and to map maxicircle pre-edited and edited transcripts and minicircle transcripts. The transcription of minicircle-encoded guide RNAs was confirmed and novel information about minicircle gene expression was obtained. The biological context involved a comparison of two strains of the parasites, one recently isolated and having an intact mitochondrial genetic system and the other an old lab strain that has developed a partially defective mitochondrial genome. The data are important for an understanding of the mitochondrial genomic complexity and expression of this dynamic genetic system.

## Introduction

Kinetoplastid protists have an unusual mitochondrial genetic architecture: the mitochondrial genes + cryptogenes encoded in the 20–40 catenated maxicircles, and multiple guide RNAs (gRNAs) encoded mainly in the ~10,000 catenated minicircles [1]. The gRNAs contain information for correction of DNA-encoded mRNA frameshifts at the RNA level [2]. This unusual genetic organization reflects the evolution in these early protists of a post-transcriptional RNA modification phenomenon known as U-insertion/deletion RNA editing. The mechanism of this type of RNA editing involves hybridization of the 5’ end of the gRNA to the “pre-edited” mRNA sequence just downstream of the first editing site, endonuclease cleavage at the first upstream mismatch, followed either by the 3’ addition of U residues to the 5’ mRNA fragment which base pair with A or G “guiding nucleotides” in the gRNA and ligation of the two RNA fragments, or removal of unpaired 3’ U residues before RNA ligation [2]. The proteins responsible for this process are components of a 1 MDa core complex (RNA editing core complex or RECC) [3] containing three endoribonucleases, two 3’-5’ U-specific exonucleases, a 3’ uridylyltransferase or TUTase and two RNA ligases, in addition to more than 10 proteins of uncertain function [4]. In addition there are a number of accessory complexes which interact dynamically with the RECC forming a yet poorly defined “editosome” complex sedimenting around 40S [5]. The accessory complexes exhibit gRNA binding [6, 7], RNA binding [8], RNA helicase [9, 10], 3’ TUTase [11–14] and poly A polymerase activities [15, 16]. Medium resolution cryoEM structures of the RECC particles from *Leishmania tarentolae* and *Trypanosoma brucei* have been published [17, 18] and several crystallographic structures of editing proteins are also known [19–26].

In *L*. *tarentolae*, there are approximately 10,000–20,000 minicircles and 20–50 maxicircles all catenated into a single giant network of DNA (kinetoplast or kDNA) [27–29] that is situated in the mitochondrial matrix linked to the flagellum basal body by fibers extending from the kDNA region to the mitochondrial membrane and from the mitochondrial membrane to the organelle (TAC complex) [30, 31]. The extent of editing varies from a few uridines at a few sites to hundreds of uridines at hundreds of sites (pan-editing). Pan-editing proceeds 3’ to 5’ with the overall polarity being determined by multiple overlapping gRNAs, one of which initiates editing of a short “block” of nucleotides, creating a sequence to which the second overlapping gRNA hybridizes, and so on, forming an editing "cascade" [32]. The mitochondrial genome of trypanosomatid protists consists of the maxicircle genome, which encodes 6 genes, transcripts of which are never edited, and 12 cryptogenes, transcripts of which are edited, and the minicircle genome, which encodes small guide RNAs (gRNAs) that contain the information for the precise insertion/deletion of uridine residues in the cryptogene transcripts to create translatable mRNAs [2, 33, 34]. Seven of the gRNAs are encoded in the maxicircle genome and the remainder are encoded in the thousands of catenated minicircles. In *L*. *tarentolae*, the minicircles are approximately 850 bp and there is a single gRNA gene per molecule. A minicircle “sequence class” is defined as a group of minicircles with homologous sequences encoding a specific gRNA. Guide RNAs which differ in sequence due to the existence of G/C and G/U base pairs but contain the same editing information are termed “redundant gRNAs”. Replication of the kDNA and the nuclear DNA is fairly synchronous in the trypanosomatid cell cycle [35, 36], giving rise to G2 networks with double the number of minicircles. Division of the kinetoplast network involves non-mitotic scission yielding two daughter networks.

The frequencies of different minicircle classes in the network of *L*. *tarentolae* were previously shown to change significantly during continuous culture from year to year [37]. The complete loss of a single minicircle class encoding a non-redundant gRNA for an essential protein would be lethal. Since division of the single kDNA network involves a non-mitotic scission of the catenated structure, it would be essential for the cell to have the minicircle classes distributed randomly throughout the network so that each daughter network contains a complete complement of gRNAs. A novel mode of DNA replication which involves decatenation of closed minicircles from the network followed by replication and then recatenation at two peripheral antipodal nodes while the network is rotating or oscillating may have evolved to help accomplish this [27]. A computer model of minicircle replication and network segregation assuming random distribution of minicircles to daughter networks and several other reasonable assumptions indicated that the frequencies of different minicircle classes spontaneously fluctuate over one thousand generations and that minicircles encoding gRNAs for non-functional editing cascades become the majority of the 10,000 molecules in the network [38]. In fact, this appears to be the case in the UC strain, in which the kDNA network was found to contain several high copy number minicircle classes that encode such “orphan” gRNAs [37]. We showed previously that the old laboratory UC strain (Tar II) of *L*. *tarentolae* isolated by Parrot in 1939 [39] was defective in editing of several of the normally pan-edited genes and therefore in the protein products which were apparently not required in culture [40]. The recently isolated LEM125 strain was found to contain minicircle-encoded gRNAs which would allow editing of a number of mRNAs not edited in the UC strain. This model however was brought into question by the finding that a strain of *L*. *mexicana* isolated in 1972 and kept in culture contained an apparently complete set of pan-edited RNAs [41], suggesting that some yet unknown property of *Leishmania* may play a role in maintaining the intactness of the editing system.

In order to understand the detailed role and plasticity of minicircle-encoded gRNAs and the details of the editing process, a complete inventory of the mitochondrial genome and the minicircle transcriptome is essential and that is presented in this paper. There is one study in the literature on the gRNA transcriptome of insect phase *T*. *brucei* [42] but no mapping was done and the minicircle genome was not investigated. In our study, minicircles from both *L*. *tarentolae* strains were sequenced and the number of classes determined. In addition, mitochondrial RNA libraries were sequenced and the reads mapped to minicircles, encoded gRNAs and pre-edited and mature maxicircle edited sequences. We confirmed and quantitated the differences between the defective kDNA genome of the UC cells and the more robust kDNA genome of the LEM125 cells.

## Materials and Methods

### Cell culture

The UC strain was originally obtained from Dr. W. Trager. It has been maintained from 1968 to the present in the L.S. lab at UCLA in Brain Heart Infusion Medium with 10 μg/ml hemin at 27°C. This is the original Parrot TarII strain (ATCC 30267, 30143) which was isolated from *Tarentola mauritanica* in Algeria in 1939. The LEM125 strain was obtained from Dr. J.A. Rioux who isolated it from a gecko in southern France in 1981 [43]. It was kept frozen in liquid nitrogen and samples were thawed to start cultures. For culture of LEM125, 10% inactivated fetal calf serum and 10 μg/ml hemin were added to BHI.

### Library preparation

Purified kinetoplast-mitochondria [44] from the UC and LEM125 [45, 46] strains of *L*. *tarentolae* were frozen and kept in Trizole. RNA was isolated using the Qiagen miRNeasy Micro Kit in which the sample homogenization in QiaZol was substituted by Trizole. RNA was fractionated into large (>200 nt) and small (<200 nt) fractions using the Ilumina RNeasy MinElute Cleanup Kit (cat. no. 74204). Small RNA libraries were generated using the SeqMatic TailorMiX miRNA sample prep kit with some modifications: The gRNA 5' triphosphate was removed by treating the RNA with Antarctic phosphatase from NEB (using NEB protocols). A 5' monophosphate was added back to the RNA for subsequent adapter ligation using T4 polynucleotide kinase from NEB (again using NEB protocols). Adapter ligation, reverse transcription, and PCR steps followed standard protocols for small RNA library generation.

Large RNA libraries were generated using the SeqMatic Directional RNA kit. The RNAs were fragmented in MgCl_2_ and the fragment ends were treated with T4 PNK. Then adapters were ligated followed by RT-PCR just as in the small RNA procedure. For sequencing using the MiSeq v2 platform [47], small RNA was run on a 150 bp single end read and large RNA on a 2x150 bp paired end read.

In one preparation, total cell RNA was used to generate libraries using SeqMatic’s TailorMix Directional RNA sample prep kit using the company’s protocols. The total RNA was sheared to an average length of 200 bp. The sheared fragments were then ligated with adapters and converted into cDNA. PCR was then performed with primers containing barcoded Illumina adapter sequences. The cDNA was then subjected to paired-end 2 X 150 bp sequencing. All libraries were validated using the Agilent Bioanalzyer.

### Contamination of libraries

The percentage of minicircle specific reads in the libraries was low, with the majority of reads specific for genomic RNAs and maxicircle rRNAs, indicating that the isolated kinetoplast fractions were contaminated with genomic RNAs. The mapping of reads to minicircles however was very specific and not affected by this contamination. In fact we were able to use libraries of reads prepared from total cell RNA and obtain quite similar results. In all mapping experiments using small RNA reads, the Bam files from reads from purified mitochondria were merged with the Bam files from reads from total cell RNA. The unmapped values for LEM small and LEM large are quite high, perhaps for technical reasons, but this does not affect our results due to the high specificity of mapping we observe. To determine contamination of libraries, RNA sequence reads were mapped to genomic, maxicircle, and all minicircle sequences in a single step. Reads were characterized by which sequence they aligned to or if they failed to align to any of these sequences. See Table 1.

**Table 1:** Contamination of libraries.

	LEM—small	UC—small	LEM—large	UC—large
Minicircles	0.17%	0.45%	0.01%	0.01%
Maxicircles	0.20%	7.36%	7.18%	19.49%
18s rRNA	10.34%	48.11%	10.79%	20.49%
Genomic	7.55%	40.96%	9.84%	22.61%
Unmapped	81.74%	3.12%	3.12%	37.40%

### Minicircle DNA sequencing

In order to obtain the complete complement of minicircle sequence classes, kinetoplast DNA was isolated by the CsCl sedimentation method [48] from stationary phase UC and LEM125 cells. The kinetoplast DNA in a microtube was cleaved with the Covaris S2 Focused-Ultrasonicator using conditions (Duty cycle 2%, Intensity 4, 200 cycles per 20 sec burst) that yielded approximately a single random cleavage per molecule. DNA libraries were generated using the Illumina TruSeq DNA sample preparation kit. All libraries were validated using the Agilent Bioanalyzer High Sensitivity DNA Assay. The average UC library size was 871 bp and the LEM library size 963 bp.

**Fig 1:**
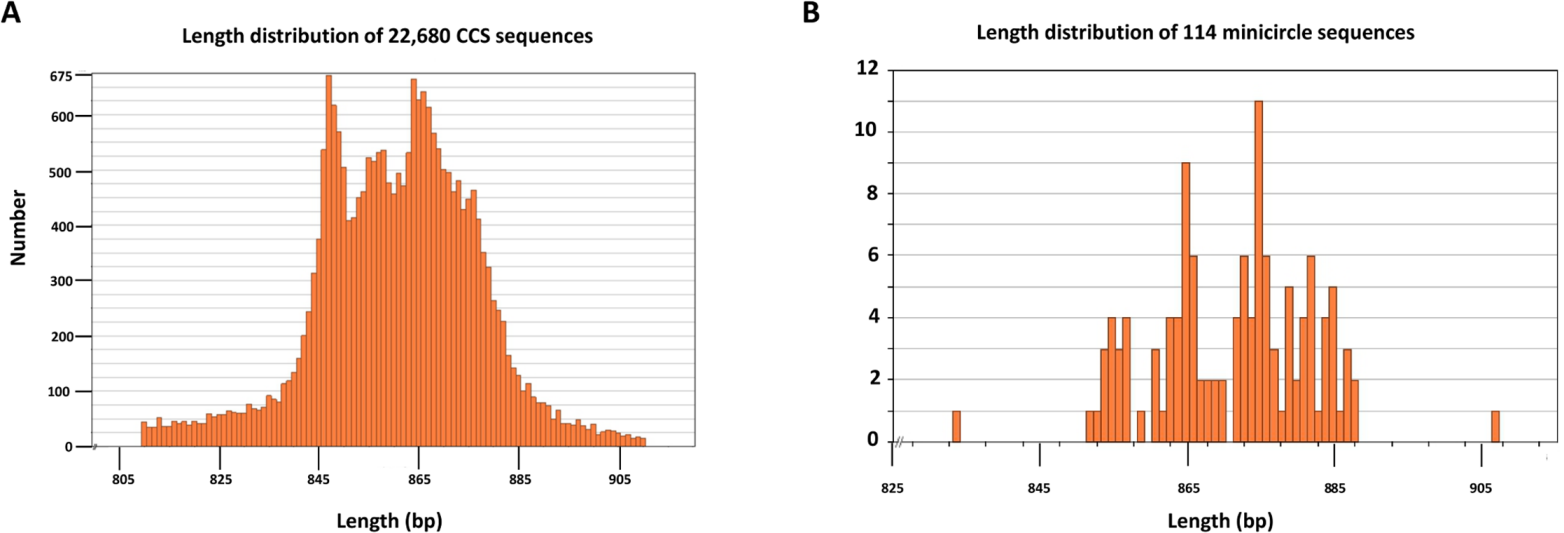
Length distribution of minicircles. A. Length distribution of 22,680 randomly single cleaved minicircle DNA molecules sequenced on PacBio platform. B. Length distribution of the final dataset of 114 minicircles.

The DNA libraries were subjected to PacBio sequencing [47] to obtain full length minicircle sequences [49] and avoid the problem of the presence of the conserved region affecting assembly of shorter sequences. CCS reads, which are circular consensus sequences generated by multiple passes around each circular SMRTbell construct, were used for minicircle sequence assembly. The CCS sequences (Fig 1A) were filtered for size (800–100 nt) and for minicircle sequences using the highly conserved CSB3 motif and subsequently rearranged in the same polarity to terminate with the 5’ end of this motif so as to have the encoded gRNAs in a 5'-3' orientation. The rearranged reads were mapped to all known minicircle sequences and unmapped reads were separated as a subgroup. A multiple alignment of the unmapped reads was performed with the MAFFT method [50] and a tree was constructed. Consensus sequences were generated based on the alignments of reads in each branch segmented from the tree. All consensus sequences were then mapped to all known minicircles and any unmapped sequences were selected as novel minicircle candidates. The novel minicircles were verified by identification of novel gRNAs as described previously and added to the database. Then the whole process was cycled with mapping all rearranged reads against this combined minicircle database until no consensus sequences could be generated.

### Minicircle copy numbers

The filtered CCS sequences of once cleaved minicircles for both strains were assembled against the 114 identified minicircles, using the Geneious program. Table 2 shows the number of CCS sequences which assembled to each minicircle sequence. These numbers were used to generate approximate minicircle copy numbers per network as described in the text.

**Table 2:** List of minicircles from both strains. Encoded gRNAs, number of CCS sequences assembled for each minicircle and the calculated number of minicircles/network, assuming 10,000 minicircles/network. UC contigs were assembled from 4370 of 5720 CCS reads. LEM125 contigs were assembled from 19,069 of 19,469 CCS reads.

		UC	LEM	UC	LEM
Minicircle number	Encoded gRNA	Number of CCS reads which assembled to minicircle	Number of CCS reads which assembled to minicircle	Minicircles/network of 10,000	Minicircles/network of 10,000
1	gCO3Ia	5	70	16	36
2	gCO3Ib	18	63	82	32
3	gCO3II	10	50	46	26
4	gCO3III		64	0	33
5	gA6Ia		73	0	37
6	gA6Ib	9	218	28	112
7	gA6II	1	145	6	74
8	gA6IIIa	6	210	29	108
9	gA6IIIb			0	0
10	gA6IIIc	55		16	0
11	gA6IV	4	174	12	89
12	gA6V	3		15	0
13	gA6VI	10	40	16	21
14	gRPS12I	10	54	20	28
15	gRPS12IIa	26	53	149	27
16	gRPS12IIb		101	0	52
17	gRPS12III	24	107	75	55
18	gRPS12IVa		1	0	1
19	gRPS12IVb	3	46	9	24
20	gRPS12Va	35	200	151	103
21	gRPS12Vb	88	205	274	105
22	gRPS12VII	3	93	26	48
23	gRPS12VIIIa		107	0	55
24	gRPS12VIIIb	12	35	104	18
25	gND3II		172	0	88
26	gND3IIIa		52	0	27
27	gND3IIIb		55	0	28
28	gND3IVa			0	0
29	gND3IVb		34	0	17
30	gND3Va		26	0	13
31	gND3Vb		122	0	63
32	gND3VIa		155	0	80
33	gND3VIb		126	0	65
34	gND3VII		44	0	23
35	gND3VIII		148	0	76
36	gND3IXa	1176	164	4884	84
37	gND3IXb	542	360	3014	185
38	gND3IXc	16		138	0
39	gND8I		52	0	27
40	gND8II		332	0	171
41	gND8III		197	0	101
42	gND8IVa		55	0	28
43	gND8IVb		44	0	23
44	gND8V		30	0	15
45	gND8VI		201	0	103
46	gND8VIIa		87	0	45
47	gND8VIIb		240	0	123
48	gND8VIII		33	0	17
49	gND8IX		152	0	78
50	gND8Xa		164	0	84
51	gND8Xb		293	0	150
52	gND8XIa			0	0
53	gND8XIb		207	0	106
54	gND8XII		99	0	51
55	gND8XIII		183	0	94
56	gND8XIV		161	0	83
57	gND9I		145	0	74
58	gND9II		196	0	101
59	gND9IIIa		106	0	54
60	gND9IIIb		39	0	20
61	gND9IVa			0	0
62	gND9IVb		15	0	8
63	gND9IVc	2	45	17	23
64	gND9V		12	0	6
65	gND9VI		162	0	83
66	gND9VIIa	1	117	9	60
67	gND9VIIb	1	3798	9	1951
68	gND9VIII			0	0
69	gND9IXa		325	0	167
70	gND9IXb		111	0	57
71	gND9X		32	0	16
72	gND9XI		335	0	172
73	gND9XIIa		459	0	236
74	gND9XIIb			0	0
75	gND9XIIIa		48	0	25
76	gND9XIIIb			0	0
77	gND9XIVa	9	151	0	78
78	gND9XIVb		113	78	58
79	gND9XV		297	0	153
80	gND9XVI		432	0	222
81	gND9XVIIIa		188	0	97
82	gND9XVIIIb		96	0	49
83	gG3I			0	0
84	gG4Ia		344	0	177
85	gG4Ib		51	0	26
86	gG4II		32	0	16
87	gG4IIIa	6	82	20	42
88	gG4IIIb	142		564	0
89	gG4IIIc	45	317	140	163
90	gG4V		75	0	39
91	gG4VIa		93	0	48
92	gG4VIb		107	0	55
93	gG4VII		323	0	166
94	gG4VIII		204	0	105
95	gG4IX		388	0	199
96	gG4X	106	246	511	126
97	gG4XI		322	0	165
98	gG4XII		65	0	33
99	gG4XIII			0	0
100	gG4XIV		417	0	214
101				0	0
102	gLmND3a			0	0
103	gLmND3b			0	0
104				0	0
105			13	0	7
106			72	0	37
107		68	70	0	36
108	gLmG4		29	0	15
109			65	0	33
110			68	0	35
111				0	0
112				0	0
113	gLmG4		78	0	40
114	gLmG4		9	0	5

### Informatics analysis

Informatics analysis was mainly performed using the Broad Institute Galaxy system [51] installed on the Hoffman2 cluster at UCLA. The short RNA FastQ files and the long RNA paired FastQ files were filtered to remove adapters and for quality. In addition, posttranscriptionally added 3' oligoU sequences in both the short and long RNA FastQ files were removed to avoid mapping problems. PolyA or Poly AU 3' sequences were not filtered since we are only interested in the mitochondrial RNA sequences. The processed RNA reads were mapped to the 114 *L*. *tarentolae* minicircle reference sequences using Bowtie [49]. Quantification and FPKM (Fragments Per Kilobase of transcript per Million mapped reads) analysis [52] were performed using in-house command line scripts created by S.D. and visualized using Excel. The average depth of coverage for the mapped gRNA peaks is ~150 fold for UC and ~50 fold for LEM125.

### Accession numbers

New minicircle sequences:

BankIt1789720 mc3 KP456020

BankIt1789720 mc4 KP456021

BankIt1789720 mc7 KP456022

BankIt1789720 mc12 KP456023

BankIt1789720 mc13 KP456024

BankIt1789720 mc14 KP456025

BankIt1789720 mc19 KP456026

BankIt1789720 mc22 KP456027

BankIt1789720 mc24 KP456028

BankIt1789720 mc28 KP456029

BankIt1789720 mc32 KP456030

BankIt1789720 mc33 KP456031

BankIt1789720 mc38 KP456032

BankIt1789720 mc41 KP456033

BankIt1789720 mc48 KP456034

BankIt1789720 mc52 KP456035

BankIt1789720 mc55 KP456036

BankIt1789720 mc59 KP456037

BankIt1789720 mc60 KP456038

BankIt1789720 mc61 KP456039

BankIt1789720 mc62 KP456040

BankIt1789720 mc71 KP456041

BankIt1789720 mc76 KP456042

BankIt1789720 mc82 KP456043

BankIt1789720 mc83 KP456044

BankIt1789720 mc86 KP456045

BankIt1789720 mc89 KP456046

BankIt1789720 mc91 KP456047

BankIt1789720 mc92 KP456048

BankIt1789720 mc98 KP456049

BankIt1789720 mc101 KP456050

BankIt1789720 mc102 KP456051

BankIt1789720 mc103 KP456052

BankIt1789720 mc105 KP456053

BankIt1789720 mc108 KP456054

BankIt1789720 mc109 KP456055

BankIt1789720 mc110 KP456056

BankIt1789720 mc111 KP456057

BankIt1789720 mc112 KP456058

BankIt1789720 mc113 KP456059

## Results

### Minicircle sequence classes in the UC and LEM125 strains of *L. tarentolae*

**Fig 2:**
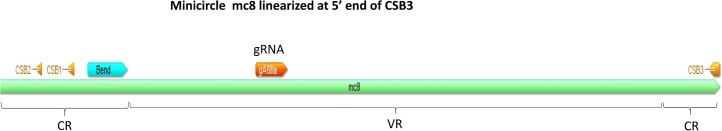
Representative linearized minicircle from L. tarentolae showing several characteristic features. The mc8 minicircle is linearized at the 5’ end of the CSB3 sequence. The “bend” region is a sequence-dependent distortion of the double helix. The encoded gRNA is annotated and is located at a characteristic distance from the CSB3. The 5'-3' polarities of the three CSB sequences, the bend and the gRNA are indicated by arrows. VR indicates Variable Region, CR indicates Conserved Region.

Sequences of once cleaved minicircles from both the UC and LEM125 strains were obtained using the PacBio platform [53] and assembled into contigs against the known minicircle database using the Geneious software package [51]. Minicircles were identified using several criteria as diagrammed in Fig 2. These criteria are size (800–1000 bp), the presence and relative location of the three short conserved sequences, CSB1 (5'-GAACGCCCCT-3'), CSB2 (5'-GAACGGGG-3') and CSB3 (5'-ATGTGGTTGGGG-3') [54] which are involved in DNA replication, and also the presence and relative location of the “bend” region [55–58]. The "Variable or Divergent Region", which represents the minicircle sequence outside the conserved region, is indicated by brackets. The intrinsically bent DNA region is located a characteristic distance from the gRNA gene (see S1 Fig for length distribution) and contains 4–5 T tracts together with variable length A and G tracts that together repeat each turn of the helix (S2 Fig).

See Fig 1A for length distributions of 22, 680 PacBio CCS sequences of once cleaved kDNA and Fig 1B for the final minicircle data set of 114 sequence classes from the LEM125 strain, of which 41 were novel, showing a peak of around 850–870 bp. Putative gRNA genes were identified by computer analysis, as discussed in Materials and Methods (see S3 Fig). The conserved location of the known gRNA genes (240–340 nt from the 5’ ends of the CSB3 sequences) assisted in this identification (S1 Fig). Table 2 has a list of the final set of 114 minicircle classes together with the”mc numbers” and the putative encoded gRNAs if known.

**Fig 3:**
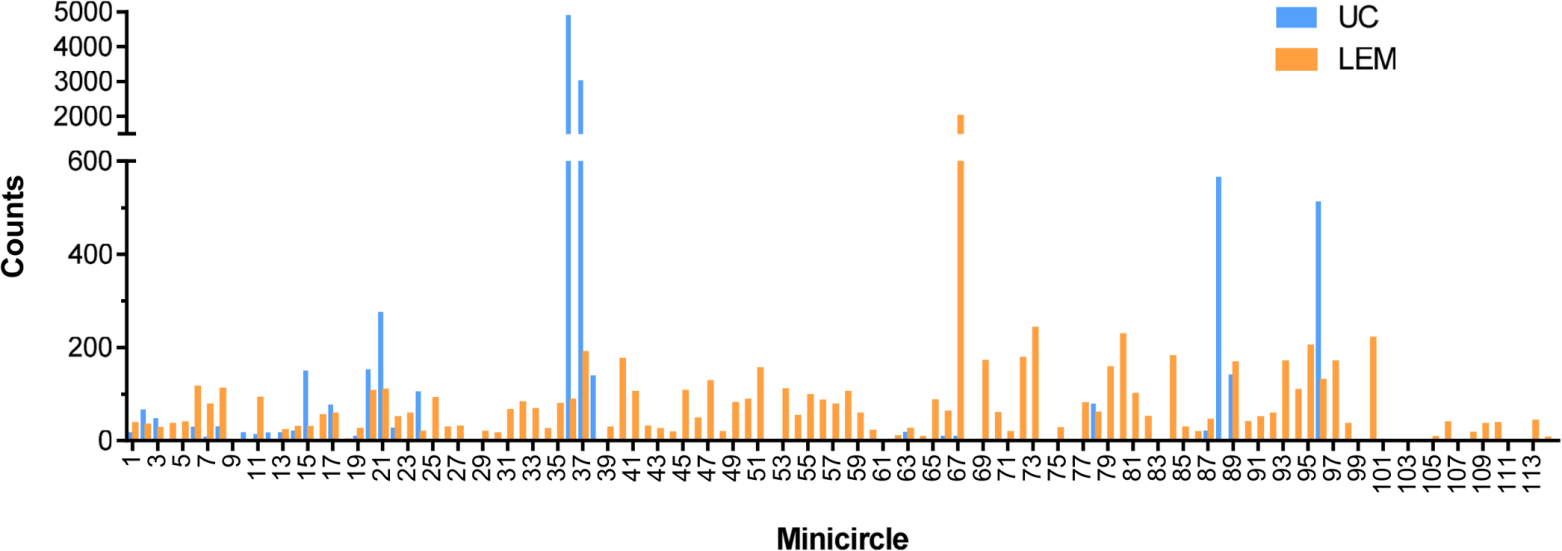
Minicircle sequence classes. The average number of minicircle sequence classes per network was calculated from the number of PacBio CCS reads that assembled to specific minicircles (see Table 2). These values are plotted for both strains as number of minicircles of a specific sequence class/network (assuming 10,000 minicircles per network) versus the mc number (see Table 2).

We define a minicircle sequence class by sequence homology and by the specific encoded gRNA. Since the kDNA network contains approximately 10,000 catenated minicircles, there must be multiple copies of most if not all of the 114 sequence classes. In order to determine the approximate frequency of the minicircle sequence classes, we assumed that the number of CCS sequences that assemble to specific minicircles is proportional to the relative frequency of these minicircles in the network. Table 2 shows the number of CCS sequences which assembled to each minicircle and the calculated copy numbers of each minicircle class for both strains. This data is shown graphically in Fig 3. The LEM125 strain has one class with ~2,000 mc’s per network of 10,000 mc's with the remainder of the 114 classes each containing less than 200 copies per network. The UC strain, however, has only 20–24 total sequence classes with two of these containing around 3,000 and 5,000 copies per network and two containing around 600 copies each.

**Fig 4:**
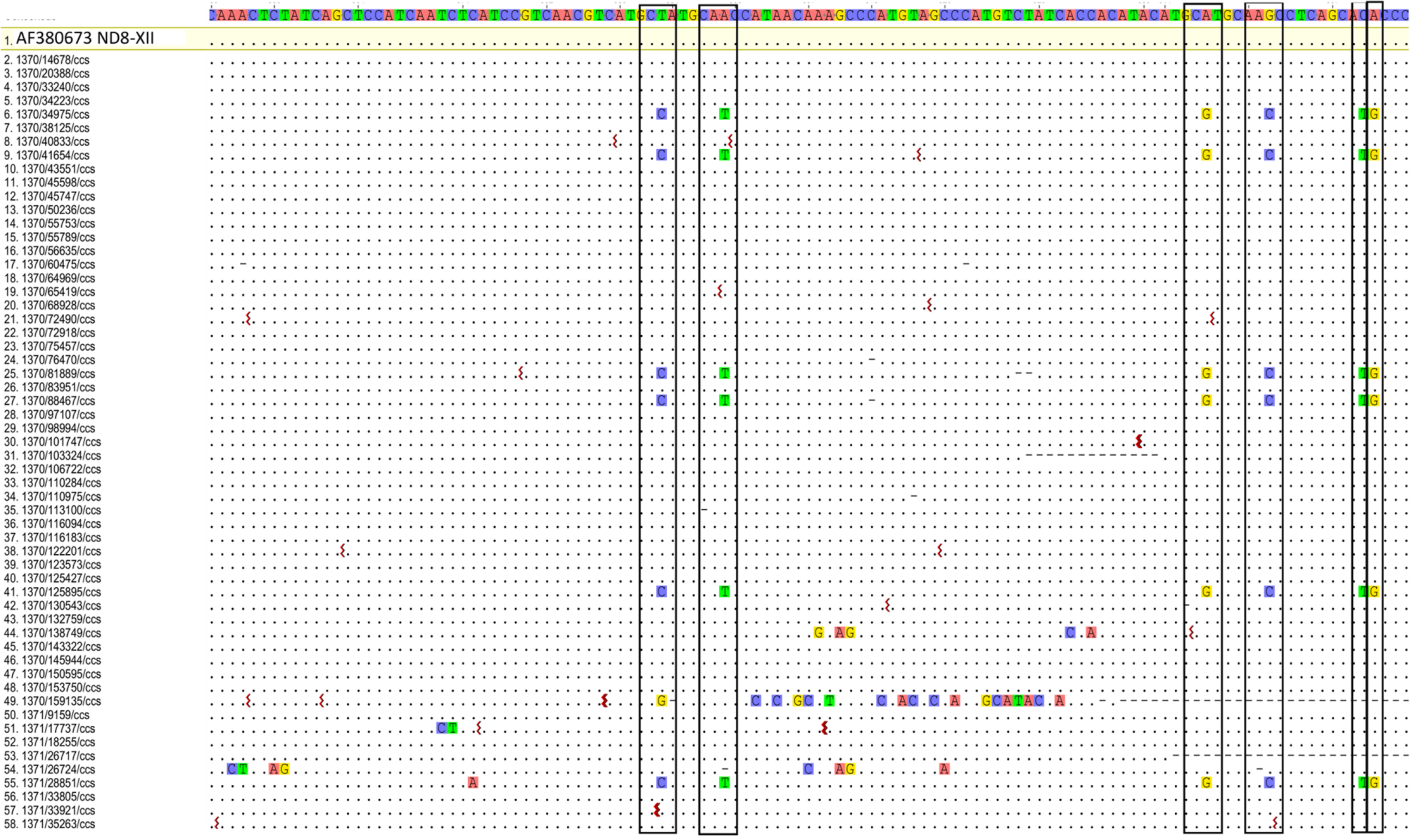
Conserved patterns of nucleotide changes. Multiple alignment of 58 CCS sequences which assembled to the minicircle, mc54, which encodes gND8-XII. The minicircle consensus sequence from nt 550–664 is at the top. Dots indicate matches and squiggles indicate deletions. Dashes indicate gaps. Columns with conserved single nucleotide mutations (T to C, A to T, A to G, G to C, C to T, A to G) in 6 of the CCS sequences at identical relative positions are boxed.

**Fig 5:**
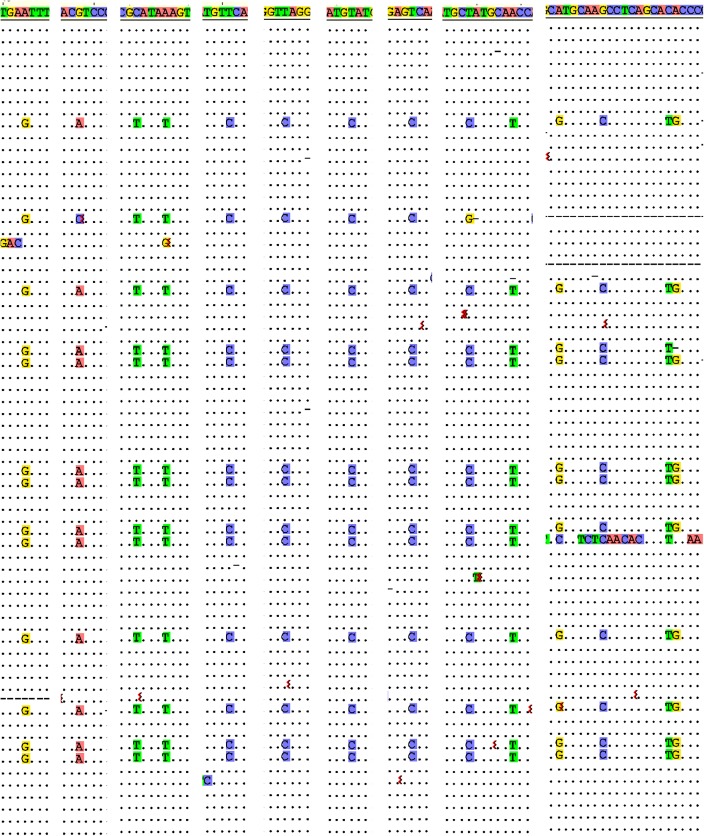
Extracted columns of conserved patterns of nucleotide changes. Nine regions were extracted from an alignment of CCS reads that assembled to a specific minicircle sequence class. Conserved patterns of single nucleotide changes in 14 columns in these regions are highlighted. See Fig 4 legend for details.

**Fig 6:**
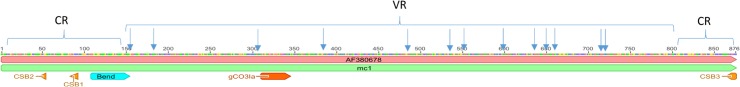
Location of conserved single nucleotide changes in representative minicircle. Diagram of linearized mc1 minicircle showing the relative locations of similar conserved patterns of single nucleotide changes (arrows) as in Fig 5. CR indicates Conserved Region, VR indicates Variable Region.

The multiply aligned CCS sequences for each sequence class showed a background of random changes, due to sequencing errors or mutations. There were also however in 61% of the sequence classes conserved patterns of single nucleotide substitutions, as shown in Fig 4 for the mc54 minicircle by the vertical boxed columns. Another example is in Fig 5 which shows 9 extracted regions from a multiple alignment of CCS sequences that form another specific minicircle sequence class. These extracted regions contain 14 columns with similar patterns of nucleotide substitutions (highlighted). Most of the conserved single nucleotide changes occur within the minicircle variable region (Fig 6).

The mutations indicated in color in Figs 4 and 5 represent highly correlated patterns. In the example in Fig 5, 14 mutations are identical in 13 CCS sequences. The probability of a nucleotide changing to another is 1/3, and the probability of identical changes in the 13 CCS sequences is (1/3)^12^. The probability of similar changes at all 14 different sites is: ((1/3)^12^)^13^ which is less than 10^−75^. In light of the extremely small probability of this happening by chance, we propose a model in which a single minicircle in a sequence class encoding a specific gRNA had multiple single nucleotide changes which were selected for perhaps by affecting transcription or processing or through some indirect affect such as growth in culture. Multiple rounds of replication of this minicircle would give rise to progeny minicircles that have the specific patterns we have observed. Further research is required to fully explain this intriguing phenomenon.

### Guide RNA genes

Putative minicircle-encoded gRNAs are identified by computer analysis, which involves searching for regions of complementarity, allowing both G-C and G-U base pairs, between the 12 mature pan-edited sequences and the minicircle sequences reversed. The duplex length criterion is >19 continuous base pairs with one or two allowed mismatches in longer duplexes due to sequence errors or to flexibility in the editing mechanism. The entire set of edited RNA/gRNA duplexes can be seen in S3–S7 Figs and the identified gRNAs are listed in Table 2.

Interestingly, there are 14 minicircles (mc101-114) which do not encode identifiable gRNAs for the *L*. *tarentolae* edited sequences. However, use of the related *L*. *mexicana* mature edited sequences led to the identification of 5 additional putative gRNAs for ND3 and G4 encoded by mc102, 103, 110, 113 and 114. The remaining 10 minicircles do not contain identifiable gRNAs.

### Locations of specific gRNAs in editing cascades for both strains and relationship to RNA editing

**Fig 7:**
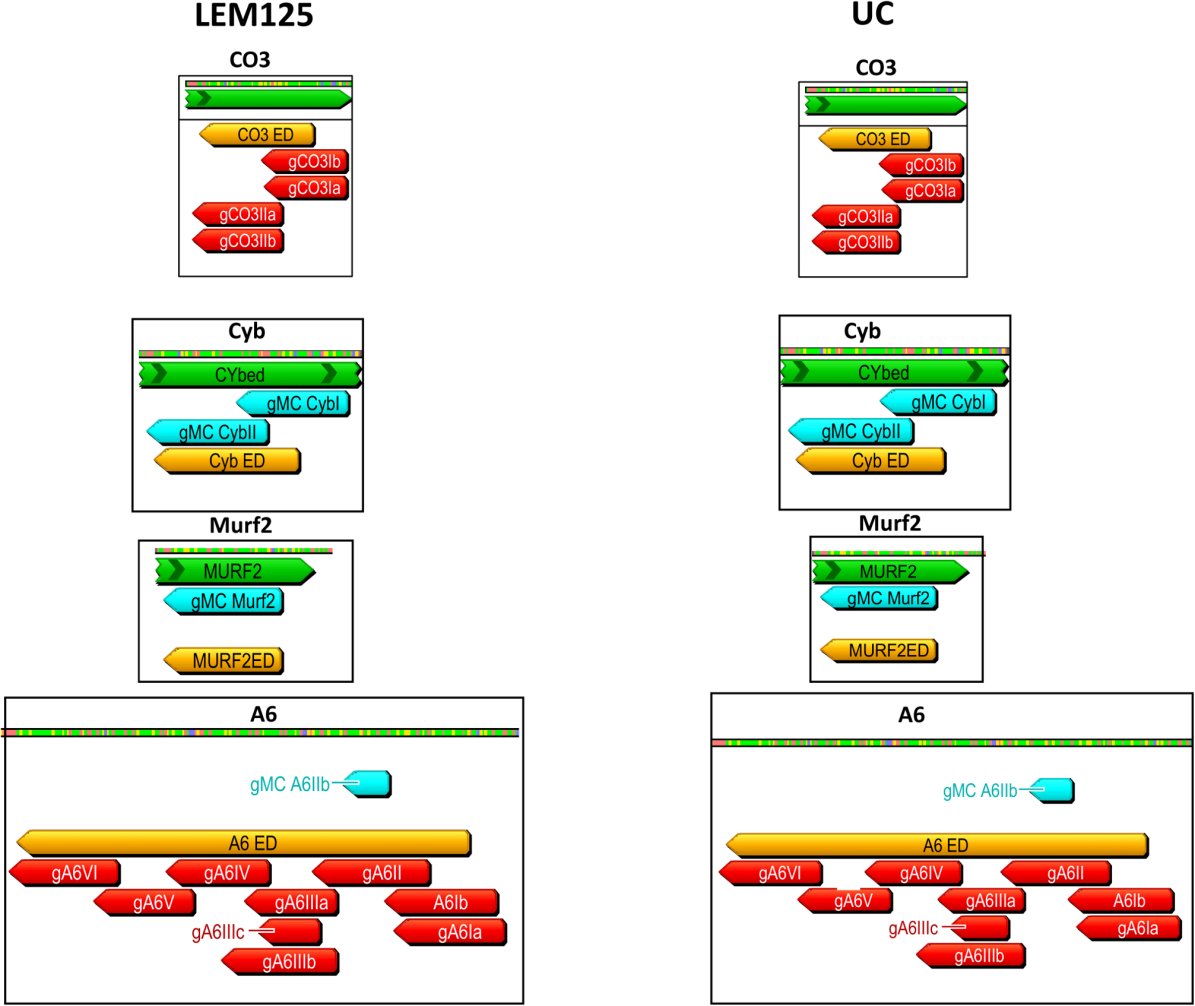
Relative positions of specific gRNAs in editing cascades for both strains. The minicircle-encoded and maxicircle-encoded gRNAs were mapped to the 12 mature edited maxicircle transcript sequences. The edited sequences (CO3, Cyb, Murf2 and A6) are in green, the editing domains are in orange and the minicircle-encoded gRNAs are in red. Maxicircle-encoded gRNAs are shown as blue arrows labeled “MC gRNA” above the sequences. The gRNAs are labeled with the gene name and Roman numerals which refer to the location in the functional editing cascade; the low numbers indicate roles early in the cascade and the higher numbers roles later in the cascade. Redundant gRNAs are indicated with a, b, etc. To easily compare the extent of gRNA coverage in the two strains, the editing domains for each gene in both strains are extracted together with the gRNA location information and shown side by side. Apparently missing overlapping gRNAs are indicated by blue circles.

**Fig 8:**
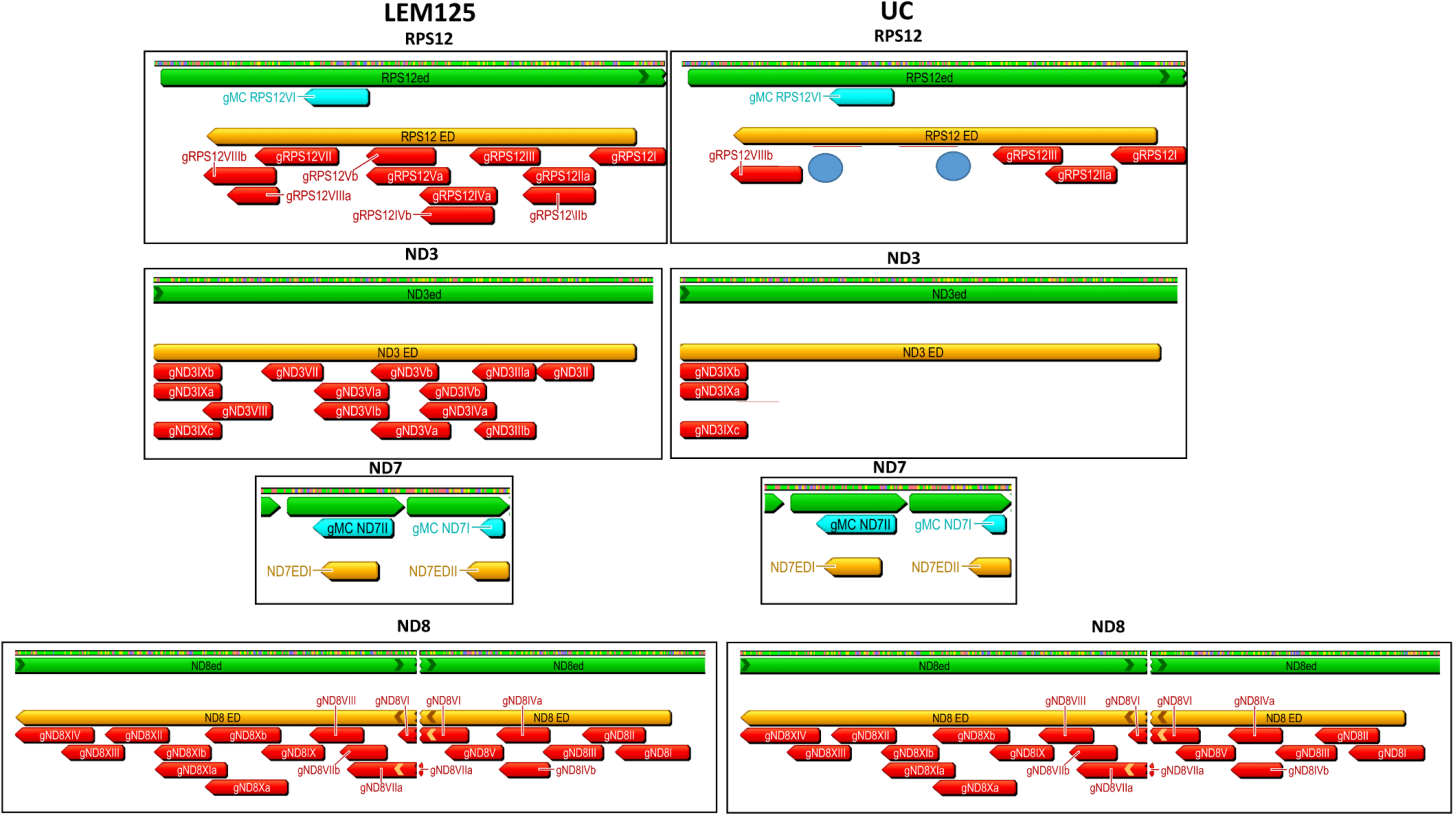
Comparison of RPS12, ND3, ND7 and ND8 genes in both strains. See Fig 7 legend for details.

**Fig 9:**
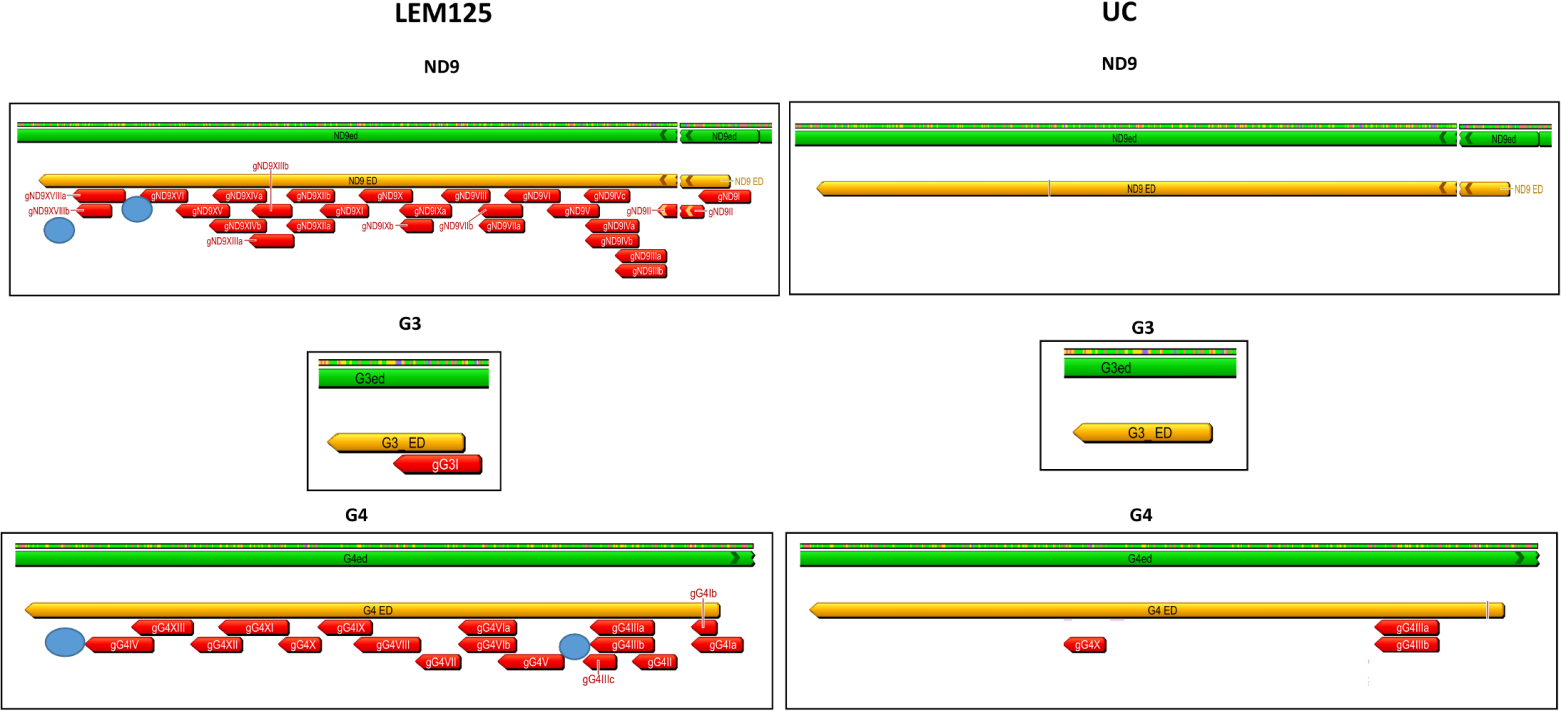
Comparison of ND9, G3 and G4 genes in both strains. See Fig 7 legend for details.

The absence of a single overlapping gRNA could break the 3'-5' editing cascade and prevent the generation of a mature mRNA and thereby translation of that product. The computer-identified gRNAs for both strains and also the maxicircle-encoded gRNAs were mapped onto the mature edited maxicircle transcripts. The extracted annotated editing domains from both strains are compared side by side in Figs 7–9. The edited sequences are in green and the editing domains in orange. The mapped minicircle-encoded gRNAs are indicated as red arrows. The 7 maxicircle-encoded gRNAs which are identical in both strains and are shown as blue arrows mediate editing of the Cyb, Murf2 and ND7 transcripts (Figs 7–9). The difference in the overall number of minicircle-encoded gRNAs between these strains is striking. LEM125 has fairly complete sets of overlapping gRNAs with multiple “redundant” gRNAs for the A6, G4, ND3 and ND8 genes (Figs 7–9). In the case of RPS12, a maxicircle-encoded gRNA covers a single gap in the cascade (Fig 8). LEM125 ND9 editing however apparently lacks at least two overlapping gRNAs (Fig 9), but we show below that the RNA library contains mature edited ND9 mRNA sequences, suggesting that our minicircle dataset lacks at least two sequence classes. LEM125 G4 editing apparently lacks two overlapping gRNAs (Fig 9) and LEM125 G3 editing lacks one gRNA required for complete editing, but we discuss below the possibility that the published 5' edited sequences for G3 may contain errors.

The UC strain on the other hand only has complete sets of overlapping minicircle-encoded gRNAs and therefore minicircle sequence classes for editing of the CO3 and A6 genes (Fig 7), and there is a striking paucity of gRNAs for editing of the ND3, ND8, ND9, G3 and G4 genes (Figs 8 and 9). UC RPS12 editing apparently lacks several overlapping gRNAs (Fig 8), but we show evidence below for the presence of mature edited UC RPS12 mRNAs, suggesting that the UC minicircle dataset lacks several minicircle RPS12 gRNA sequence classes.

### Mapping of mitochondrial RNA reads to the LEM125 and UC minicircles

**Fig 10:**
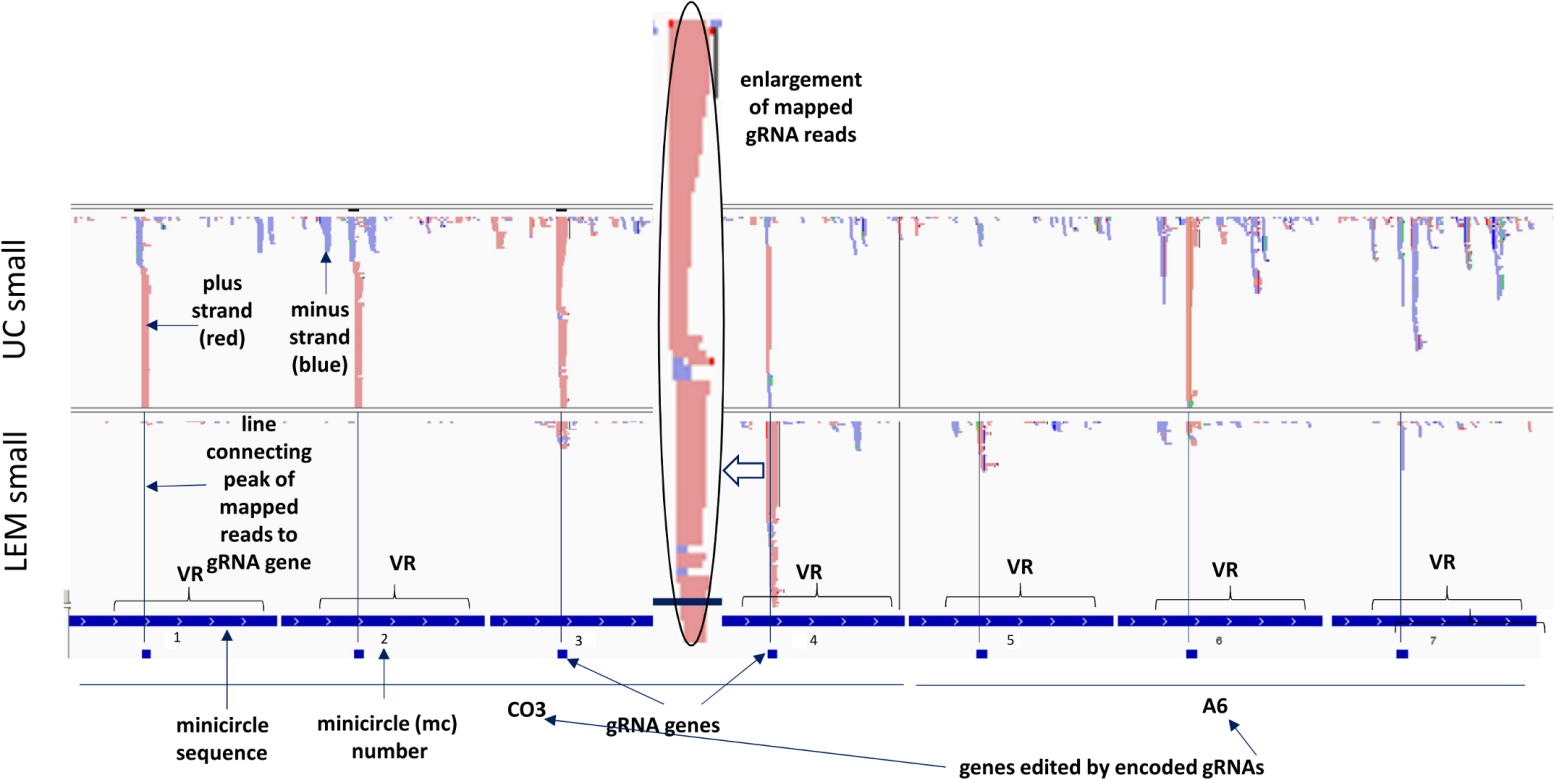
Mapping of RNA reads from both strains to the 114 minicircles. Reads from the small mitochondrial RNA libraries were mapped against minicircle sequences using Bowtie. The linearized minicircles from both strains are concatenated head to tail with 20 nt spaces inserted for the mapping and indicated under the maps by dark blue tracts with the mc numbers. The locations of the putative encoded gRNA genes within the minicircles are also annotated by small blue boxes below each minicircle tract. VR refers to the “variable region” of the minicircle. The minicircles are organized in groups of cascades according to the specific gene, the editing of which is mediated by the gRNA, and each minicircle is denoted by the “mc number”. The plus strand is light red and the minus strand blue. Lines were inserted to show that the putative gRNA genes line up with the corresponding peaks of gRNA reads. One gRNA peak (indicated by open arrow) was enlarged for better visualization of the flush 5’ ends and the heterogeneous 3’ ends.

In order to verify transcription of the putative gRNA genes, libraries for NGS sequencing were constructed for small and large mitochondrial RNA fractions (<200 nt and >200 nt) as described in Materials and Methods. These reads were used to map onto all known minicircle sequences. All minicircle sequences are in the same polarity with the gRNAs in the 5' to 3' orientation. The encoded gRNAs are annotated below each respective minicircle as small boxes. The linearized minicircles are concatenated in tandem in an order determined by the positions of the various gRNAs in the editing cascades starting with the 3' most gRNAs. A 40 nt filler nucleotide sequence separates each minicircle. Several representative minicircle mapping images are shown in Fig 10 and the entire dataset can be seen in S8–S11 Figs. Also, see S17–S19 Figs for the complete dataset of mapping of short RNA reads from both strains to the 100 gRNA sequences directly.

In Fig 10, minicircles mc1-mc4 encode gRNAs involved in the CO3 cascade of editing and minicircles mc5—mc7 encode gRNAs involved in the A6 cascade of editing. The reads are mapped using Bowtie to the concatenated linearized minicircle sequences. Lines are drawn from each encoded gRNA to indicate the putative mapped gRNA peak. The sizes of the mapped peaks indicate the number of reads that map to each region. It should be noted that in IGV the scales are automatically sized to fill the field. The variable region of each minicircle which contains the gRNA gene is annotated.

Interestingly, there are additional peaks, both sense and antisense, not coincident with the gRNA genes and not conserved between minicircles. There are no striking differences in abundance of the steady state transcripts from either strand in the minicircle variable (VR) or conserved regions.

**Fig 11:**
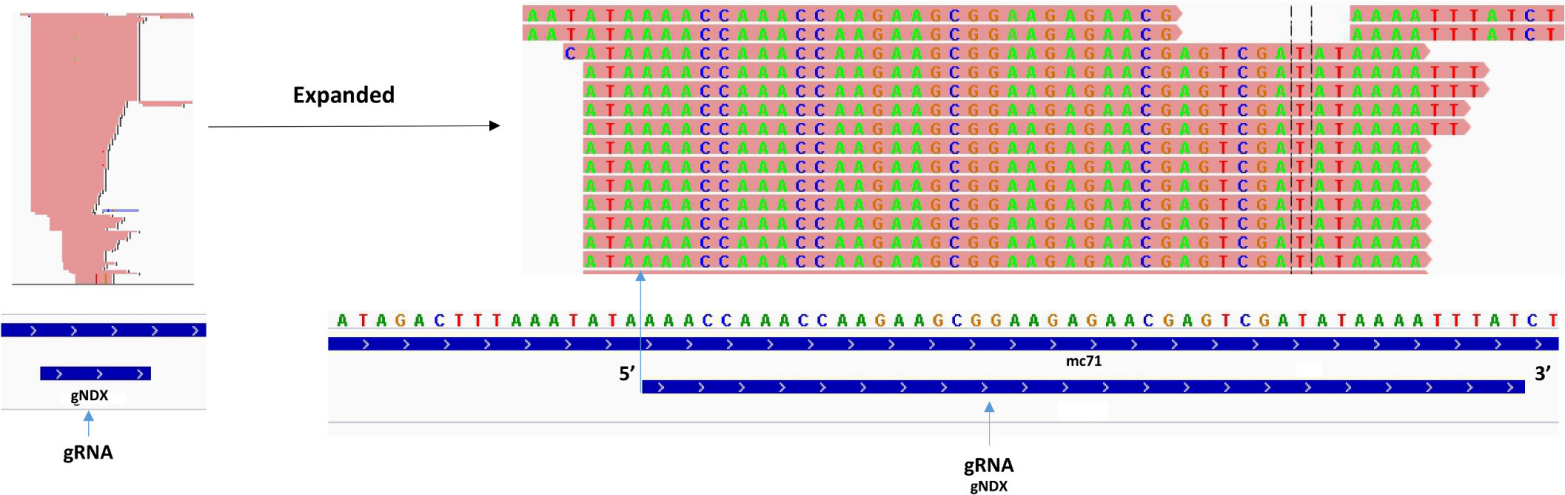
Expansion of mapped gRNA peak to nucleotide level. A mapped gRNA peak from mc71 encoding the gND9X gRNA is expanded to the nucleotide level to illustrate the characteristic 5’ flush edge and 3’ heterogeneity. Note that the major 5' start site is located 3 nt upstream of the 5' end of the identified gRNA/edited mRNA duplex. See Fig 10 legend for details.

The mc4 mapped gRNA peak is shown expanded in Fig 10 to illustrate the fairly homogeneous 5’ ends and the 3’ end heterogeneity. The gND9X gRNA mapped peak is shown expanded to the nucleotide level in Fig 11 to illustrate that the mapped gRNA reads initiate 3–4 nucleotides upstream of the beginning of the gRNA/mRNA complementarity. In some cases however the mapped gRNAs initiate a few nucleotides downstream of the gRNA/edited mRNA duplex start, but there is an overall preference for transcription initiation at the -3 and -4 positions, which are mainly A and C. In most cases due to the 3’ heterogeneity, the reads only map to a portion of the 3’ end of the gRNA gene. See S12–S16 Figs for multiple examples.

**Fig 12:**
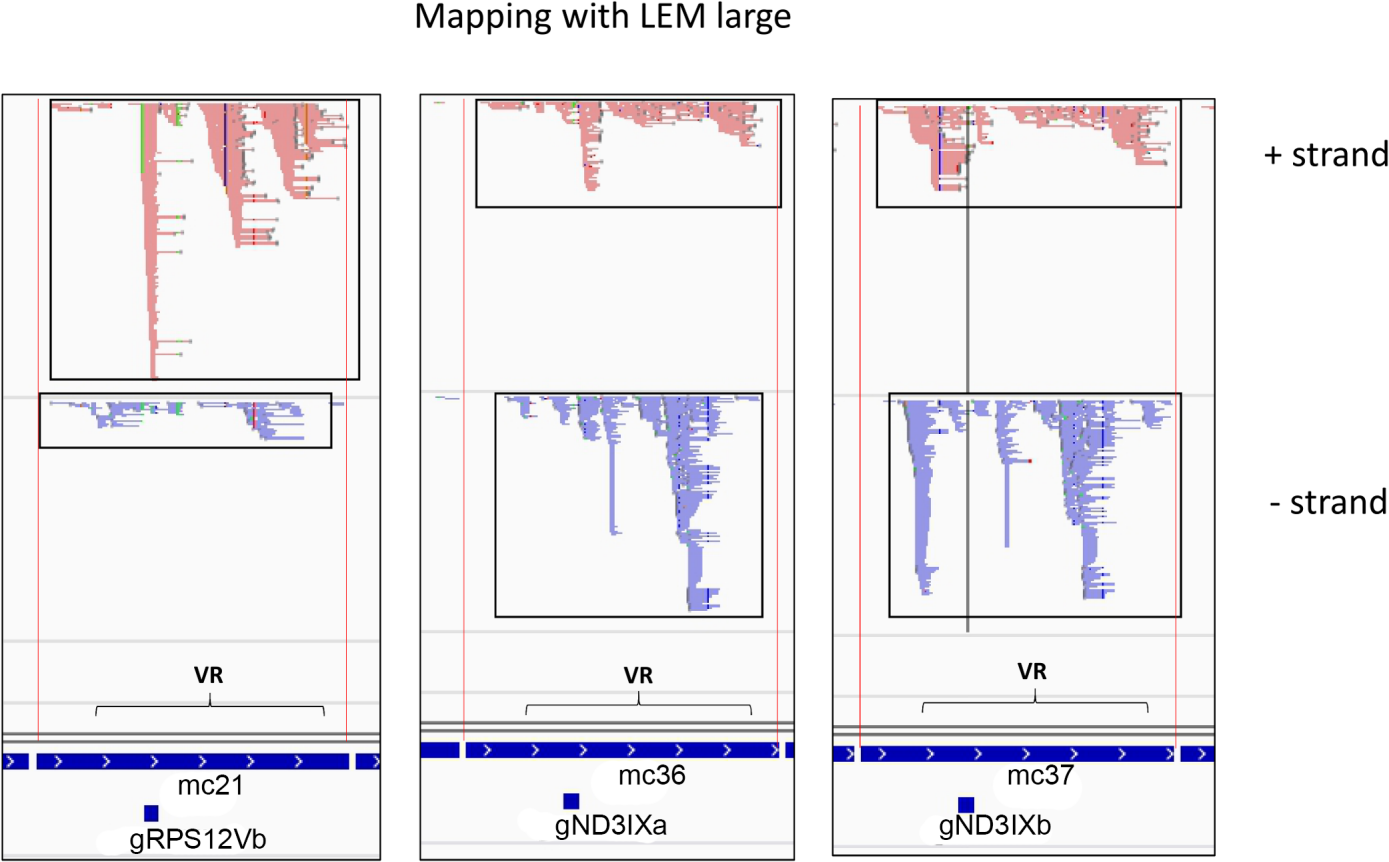
Three representative minicircles mapped with LEM large reads. The light red is + strand and the blue is—strand. The extensive mappings of steady state transcripts from both strands over almost the entire minicircle are shown boxed. See Fig 10 legend for details.

The large RNA reads in many cases cover large portions if not the entire minicircles on both strands, as shown for three representative minicircles in Fig 12. The boxes in Fig 12 show the extensive expression of both strands covering almost the entire minicircle. This data suggests that transcription is bidirectional and complete, and, since the 5' end of the mature gRNAs is an unprocessed triphosphate [2], that 3' end processing is required for maturation of the gRNAs.

Since any 3' oligo U tails were filtered from the Fastq sequences prior to mapping, the percent of bona fide gRNAs mapping cannot be determined, and it is possible that some of the mapped reads are derived from small RNAs that lack the 3' oligo U tail. This requires further investigation.

### Some minicircles do not encode gRNAs

**Fig 13:**
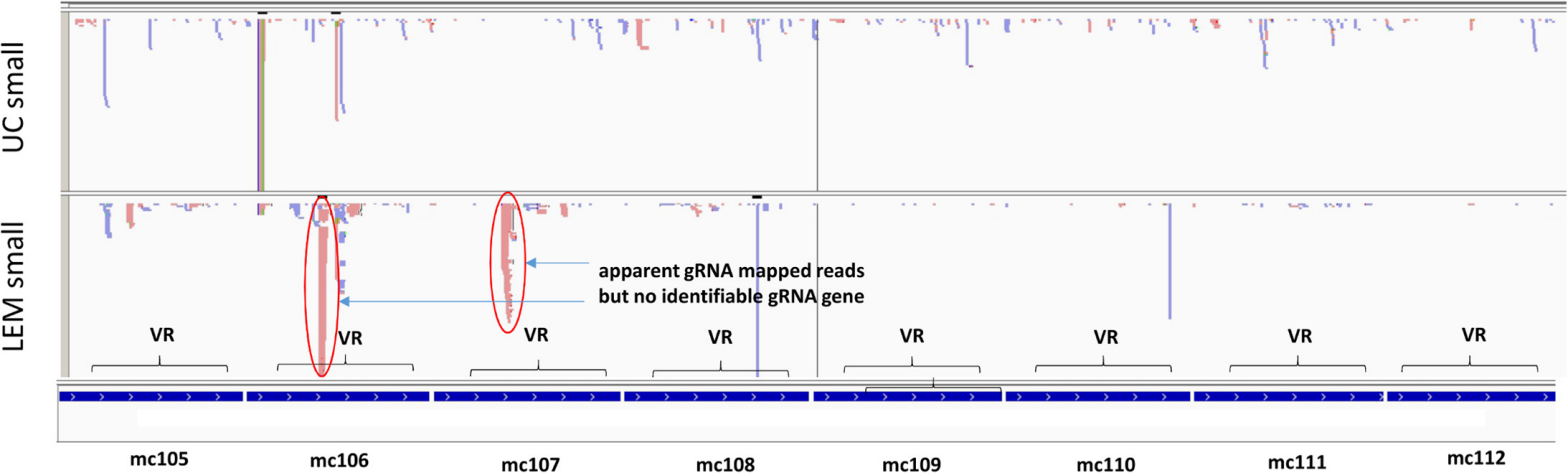
Minicircles with no identified gRNAs. Maps of 8 of the 14 minicircles (mc105-mc112) lacking identified *L. tarentolae* gRNAs are shown. The enigmatic gRNA-like mapped peaks in two of the minicircles are circled in red. See Fig 10 legend for details.

gRNA/mRNA duplex motifs for the *L*. *tarentolae* edited sequences could not be detected by computer analysis of mc101-mc114. However, putative gRNAs were identified in several of these minicircles for *L*. *mexicana* ND3 and G4 genes (mc102, 103, 110, 113, 114). As shown in Fig 13, in several minicircles without identifiable gRNAs (mc101, 104, 106, 107), peaks of mapped reads showing characteristics of gRNA peaks are present in the 240–350 bp regions (circled in Fig 13 for mc106 and mc107), but analysis of the consensus read sequences of these peaks against mature edited mRNA sequences does not yield any gRNA/edited mRNA duplexes. The function of these peaks of reads is unknown. One possibility is that they encode gRNAs for misedited [59], partially edited [59] or alternatively edited [60] RNAs, but this was not investigated. The apparent absence of gRNA genes in some minicircles is a novel and puzzling finding which should be investigated further.

### Minicircle copy number and minicircle RNA

**Fig 14:**
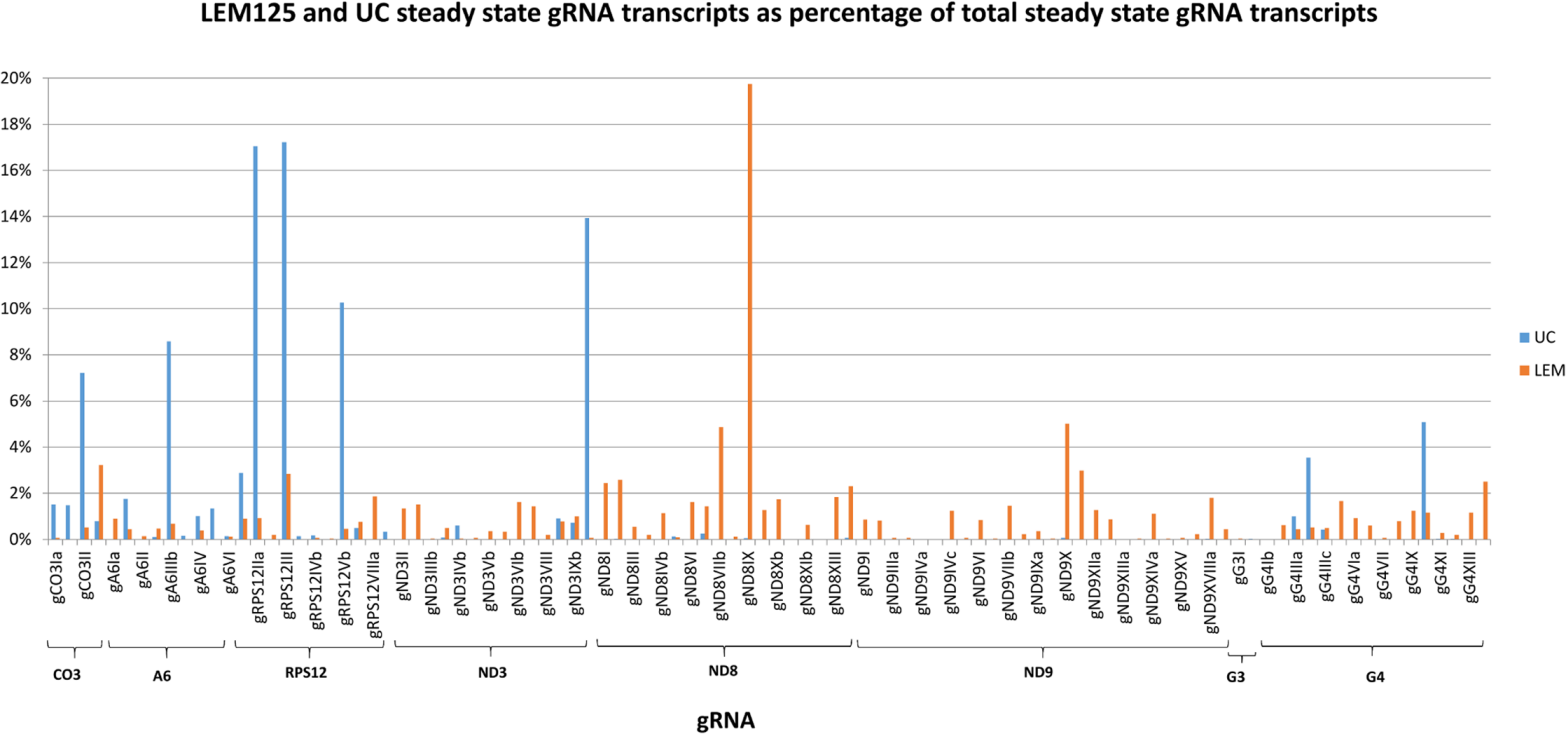
FPKM quantitation of minicircle steady state transcripts. LEM125 (light red) and UC (blue) minicircle steady state transcripts as percentage of total minicircle steady state transcripts. The minicircle numbers are shown below and the minicircles are grouped according to the gene the editing of which is mediated by the encoded gRNAs.

**Fig 15:**
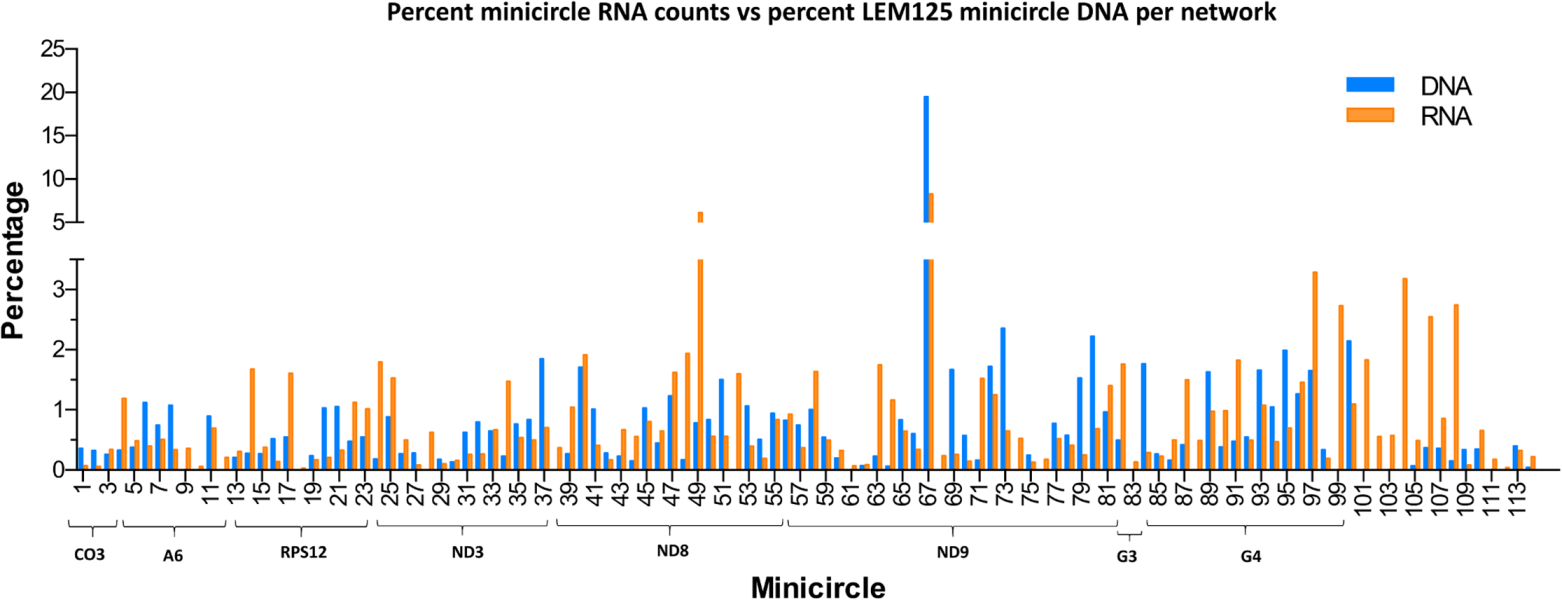
FPKM quantitation of minicircle steady state transcripts. LEM125 and UC steady state gRNA transcripts as percentage of total steady state gRNA transcripts. The names of the gRNAs are shown below and the gRNAs are grouped as in Fig 14.

The FPKM method was used to quantitate the mapped steady state expression products. Fig 14 shows the expression of specific total minicircle steady state transcripts as a percentage of total minicircle expression for both strains. Fig 15 shows the expression of specific gRNA steady state transcripts as percentage of total gRNA transcripts. There is as much as a 10 fold variation in the abundance of steady state transcripts between different classes in both strains, but there is no correlation between relative abundance of transcripts and minicircle function such as location of the gRNA in the editing cascades. In general the UC strain shows a higher relative abundance of transcripts than the LEM125. The gRNA distribution in Fig 15 differs significantly from the minicircle transcript distribution in Fig 14, which could be a function of rates of transcription and turnover.

**Fig 16:**
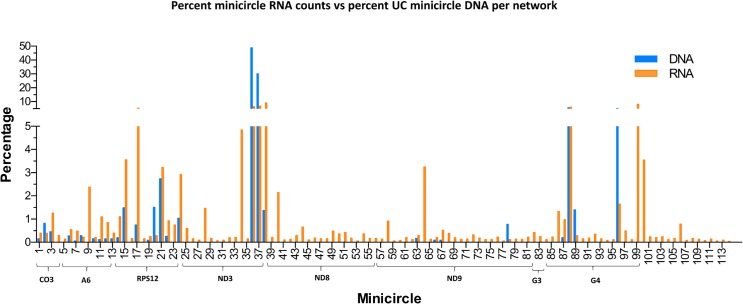
FPKM quantitation of minicircle steady state transcripts. Percent expression of steady state total minicircle transcripts compared to percent of LEM125 minicircle DNA per network. Minicircle percent data from are Fig 3. See Fig 14 legend for details.

**Fig 17:**
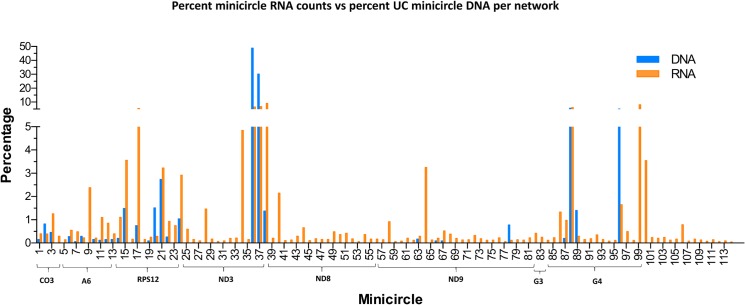
FPKM quantitation of minicircle steady state transcripts. Percent expression of steady state gRNA transcripts compared to percent of UC minicircle DNA per network. Minicircle percent data are from Fig 3. See Fig 14 legend for details.

**Fig 18:**
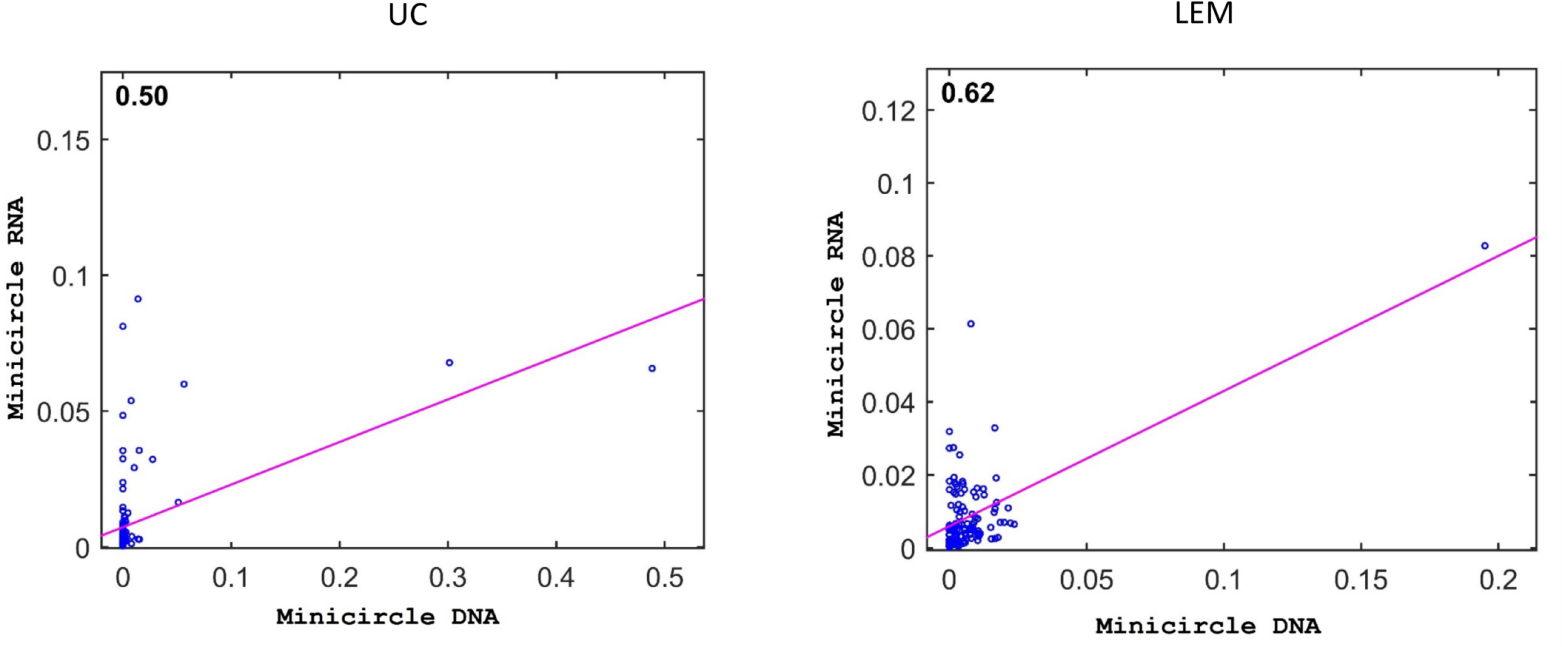
Scatter plots of minicircle steady state RNA versus minicircle copy number for LEM and UC strains. Each minicircle shown in blue with fit line in pink. R = 0.62 for LEM, R = 0.50 for UC.

One possibility for these observations might be the minicircle copy number differences. The data in Figs 16 and 17 compare the percent expression of total minicircle and gRNA steady state transcripts to the percent of the different minicircle sequence classes in the network for both strains. The most abundant LEM125 minicircle class (mc67) shows the largest extent of expression, but the second most abundant minicircle class (mc49) shows relatively little expression. In general, LEM125 minicircle copy number and LEM125 (Fig 16) minicircle expression of steady state transcripts show a moderate positive Pearson correlation [61] (r = 0.6233) while the correlation in the case of the UC strain (Fig 17) is somewhat weaker (r = 0.4971). Scatter plots of this data are shown in Fig 18.

**Fig 19:**
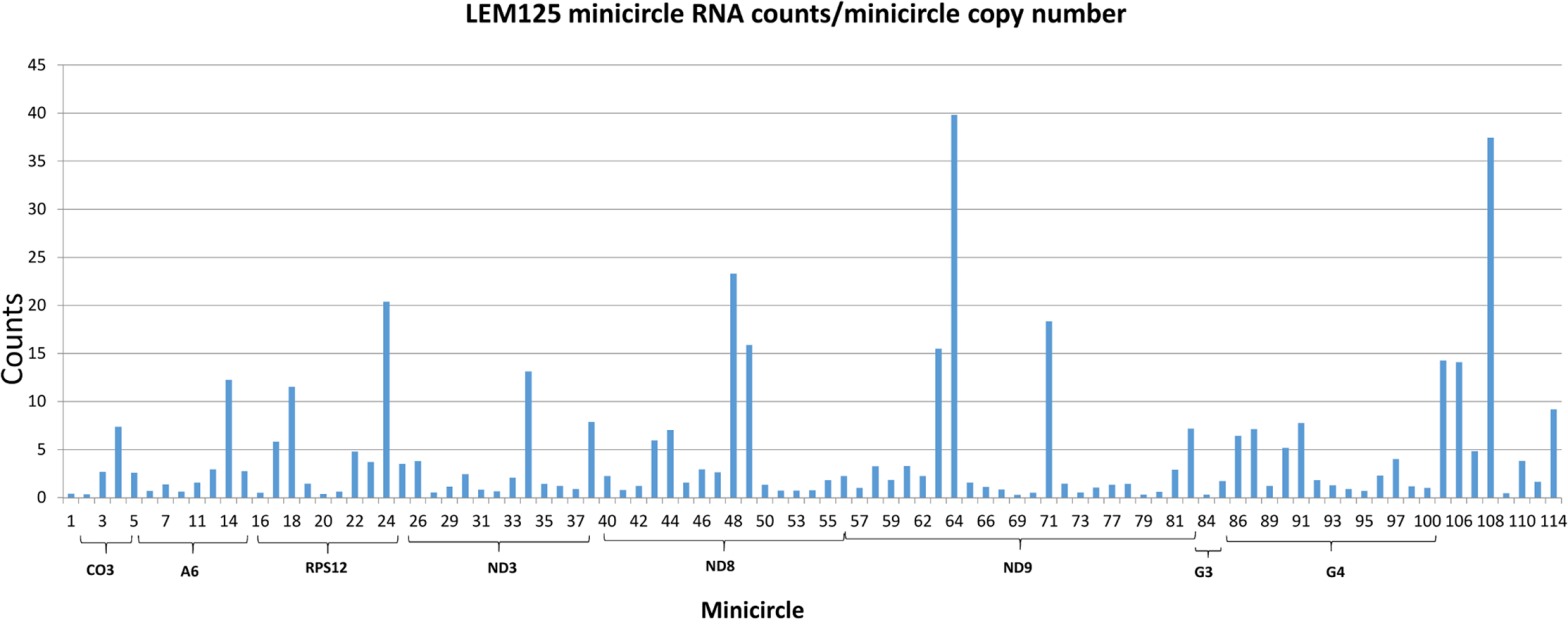
LEM125 total minicircle RNA reads divided by minicircle copy number per network. See Fig 14 legend for details.

**Fig 20:**
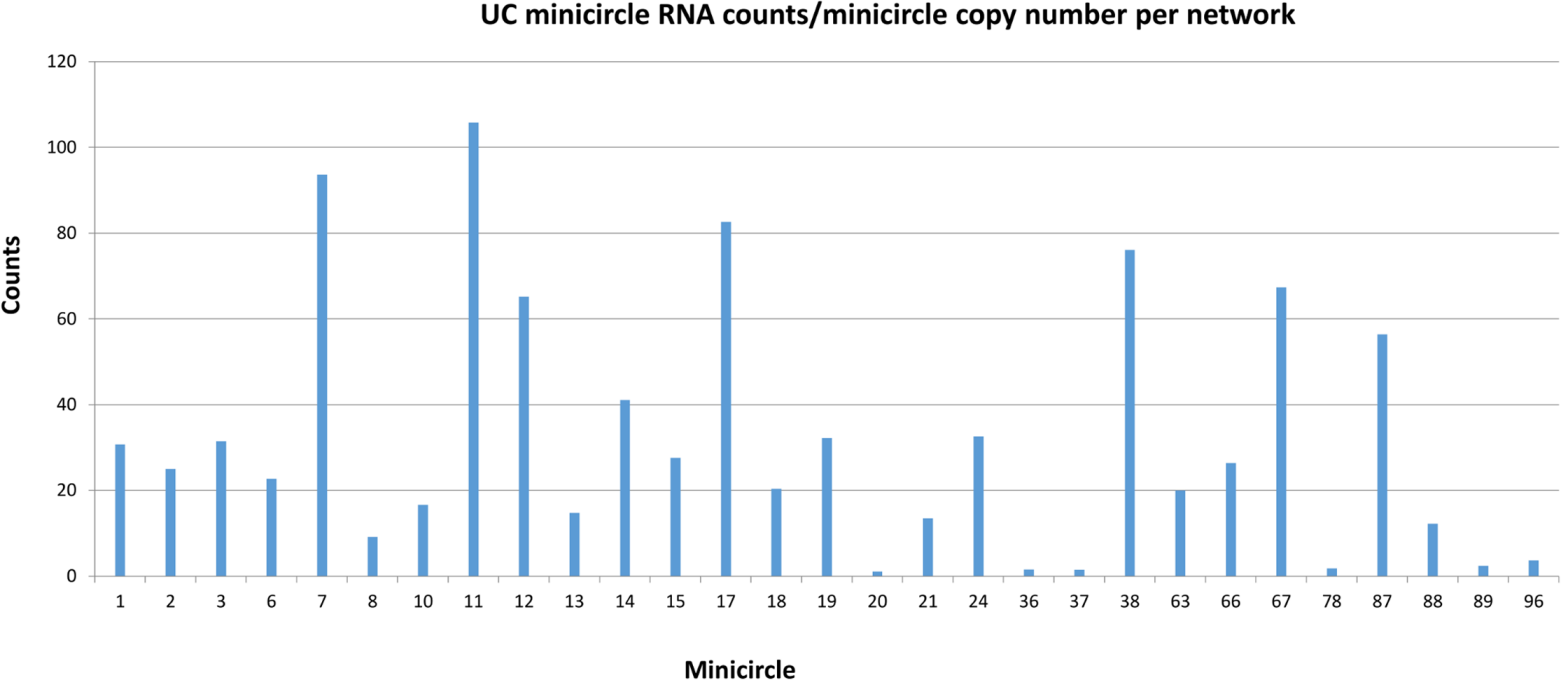
UC total minicircle RNA reads divided by minicircle copy number per network. See Fig 14 legend for details.

**Fig 21:**
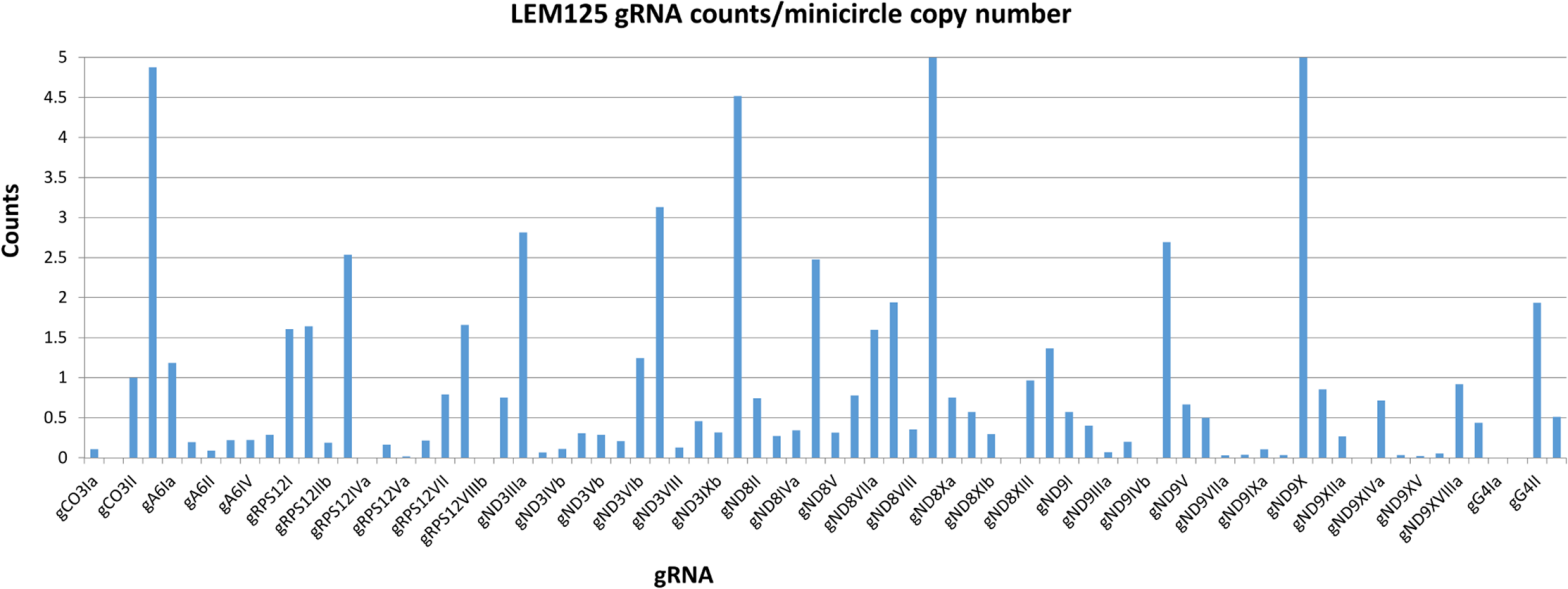
LEM125 steady state gRNA reads divided by minicircle copy number per network. See Fig 14 legend for details.

**Fig 22:**
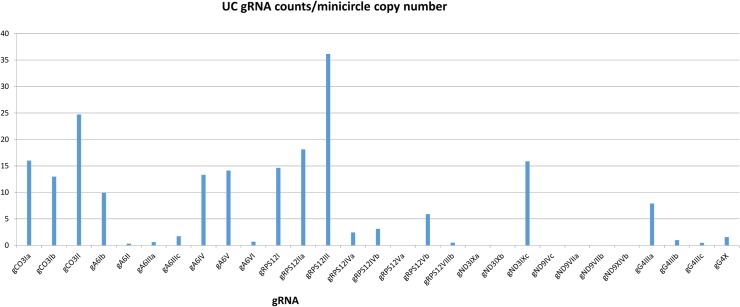
UC steady state gRNA reads divided by minicircle copy number per network. See Fig 14 legend for details.

Another possibility is that individual minicircles in different sequence classes may differ innately in expression. Fig 19 shows the normalized total minicircle steady state transcript abundance divided by the LEM125 minicircle copy number and Fig 20 shows the same data for the UC minicircles. In both there is a large variation in transcript abundance for different minicircles and there is no apparent gRNA functional correlation. A similar analysis of expression of the normalized steady state minicircle gRNA is shown in Fig 21 for LEM125 and Fig 22 for the UC strain.

### Expression of the maxicircle genome

**Fig 23:**
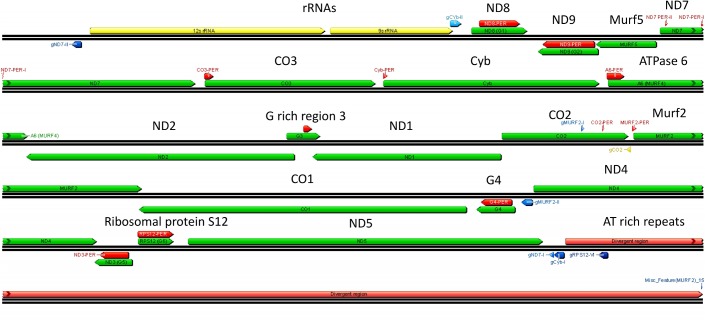
*L*. *tarentolae* maxicircle genome. Ribosomal RNAs are in yellow, structural genes and cryptogenes are in green, divergent (variable) region is in pink. Maxicircle-encoded gRNAs are in blue. Polarity is indicated by the arrow (right = plus strand, left = minus strand). “PER” indicates the pre-edited regions.

Maxicircle genes are encoded on both strands and some genes overlap (Fig 23). Seven gRNAs are also encoded in the maxicircle (blue arrows) (eight including the CO2 *in cis* gRNA sequence). There is also a large variable or divergent region with AT-rich repetitive sequences that vary in size and sequence between trypanosomatid species and the function of which is unknown.

**Fig 24:**
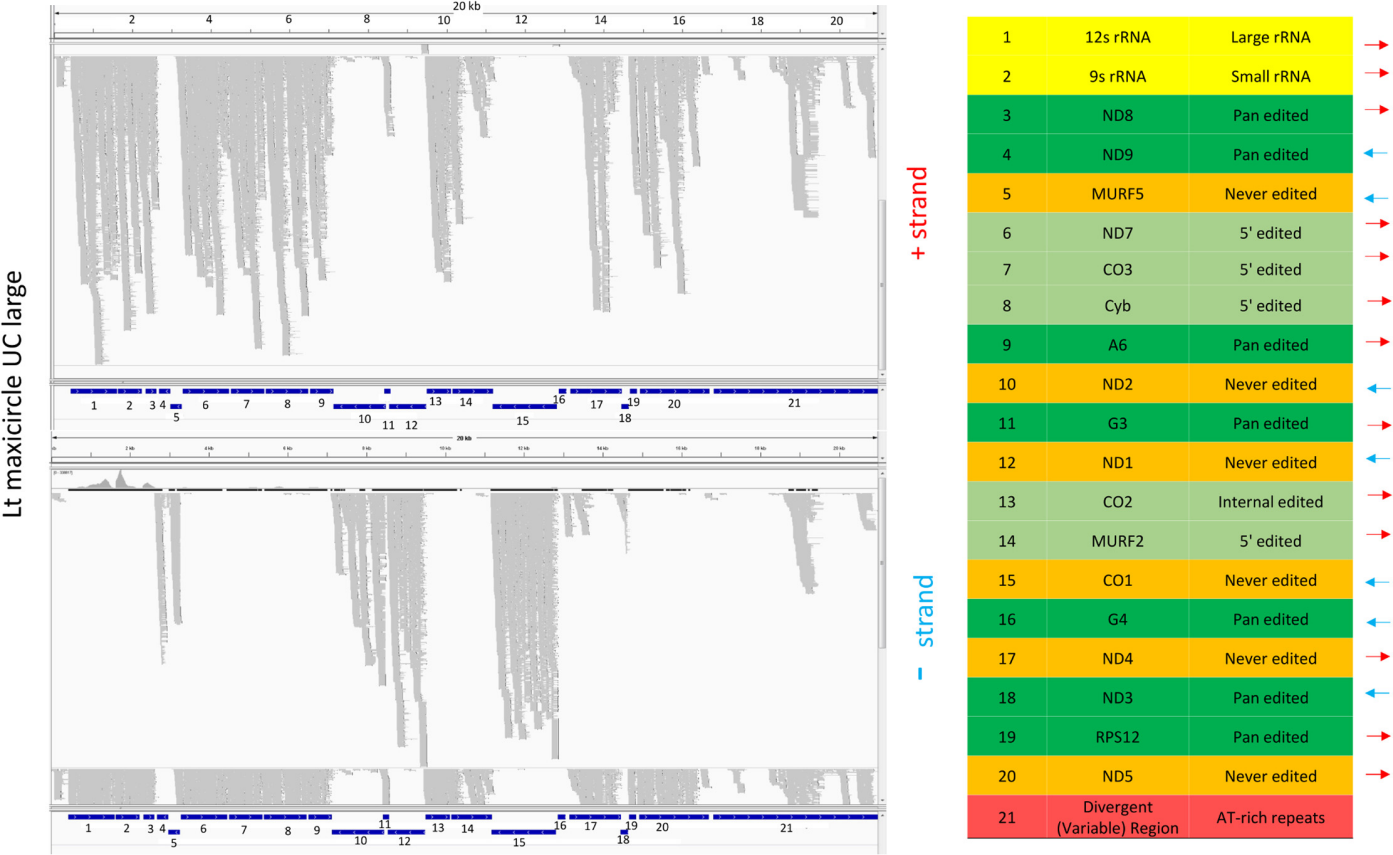
Mapping of LEM125 large RNA reads to the maxicircle genome. The plus strands are in the upper panel and the minus strands in the lower panel. The location and polarity of each gene are indicated in the table on the right. The different classes of genes are color coded: rRNAs are in yellow, never-edited genes are in orange, pan edited genes are in dark green, 5' edited genes are in light green, Divergent Region is in red. Note that the never edited genes are in orange rather than green as in Fig 23.

**Fig 25:**
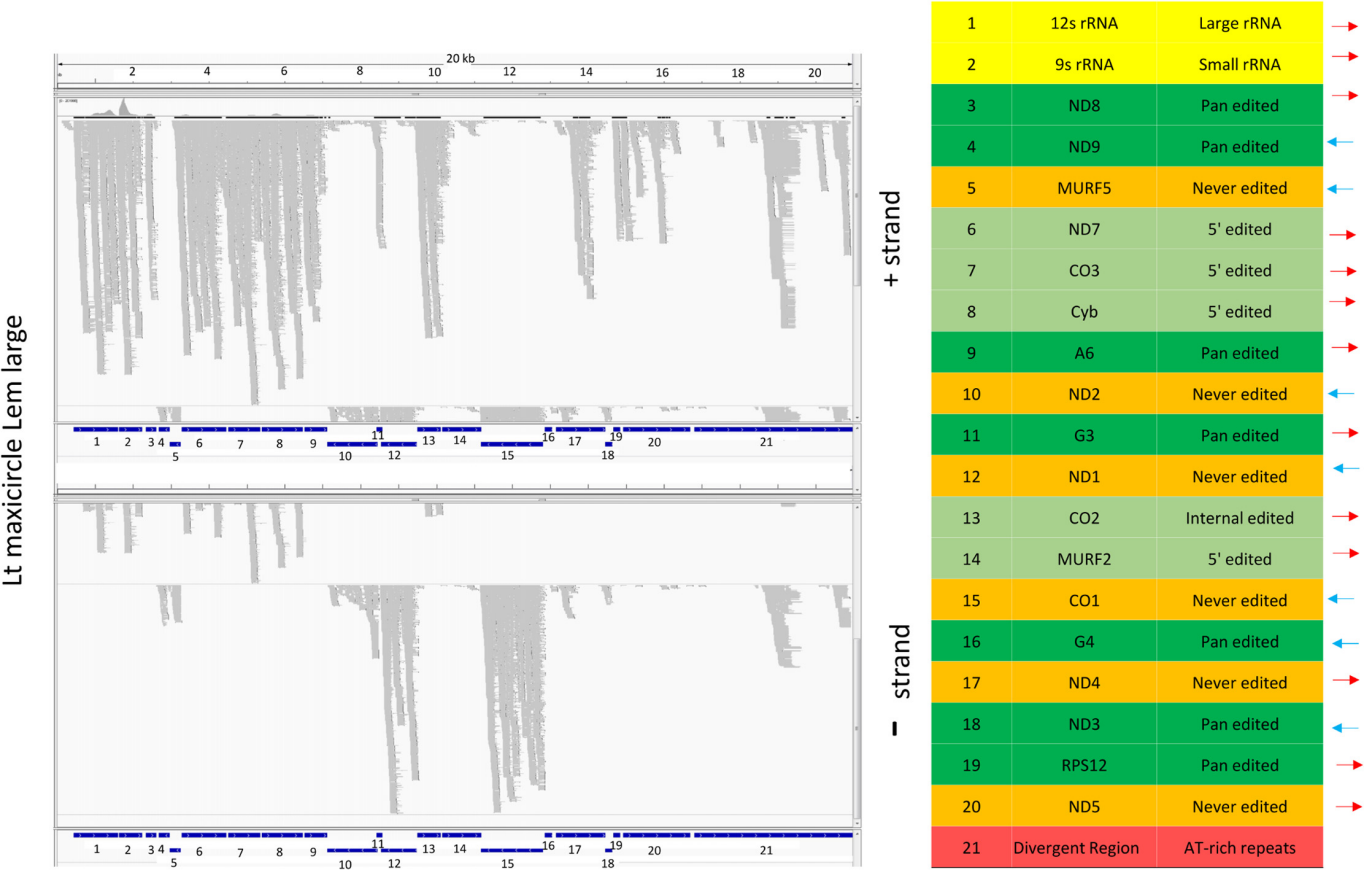
Mapping of UC large RNA reads to the maxicircle genome. See Fig 24 legend for details.

Twelve of the 18 pre-edited structural genes are edited post transcriptionally and termed “cryptogenes”, and the remainder are termed “never edited”. Both are shown in green in Fig 23 and the never edited genes are in orange in the right panels in Figs 24 and 25.

Little is known about transcription of maxicircle genes, other that there are some polycistronic transcripts which must be processed [62, 63]. Transcription of the gRNAs has the additional complication in that the gRNAs encoded in the maxicircle have 5’ triphosphates, as in the case of minicircle-encoded gRNAs [2] and may represent primary transcripts, but promoters have not been identified 5' of the gRNA genes.

Large RNA libraries from both strains were mapped to the maxicircle genomic sequence. As shown in the example in Fig 24 and Fig 25, there are no significant differences between the mapping of steady state RNAs between the strains. The polarity and identity of each gene are shown on the right.

### Mapping of RNA reads to pre-edited and mature pan-edited genes

**Fig 26:**
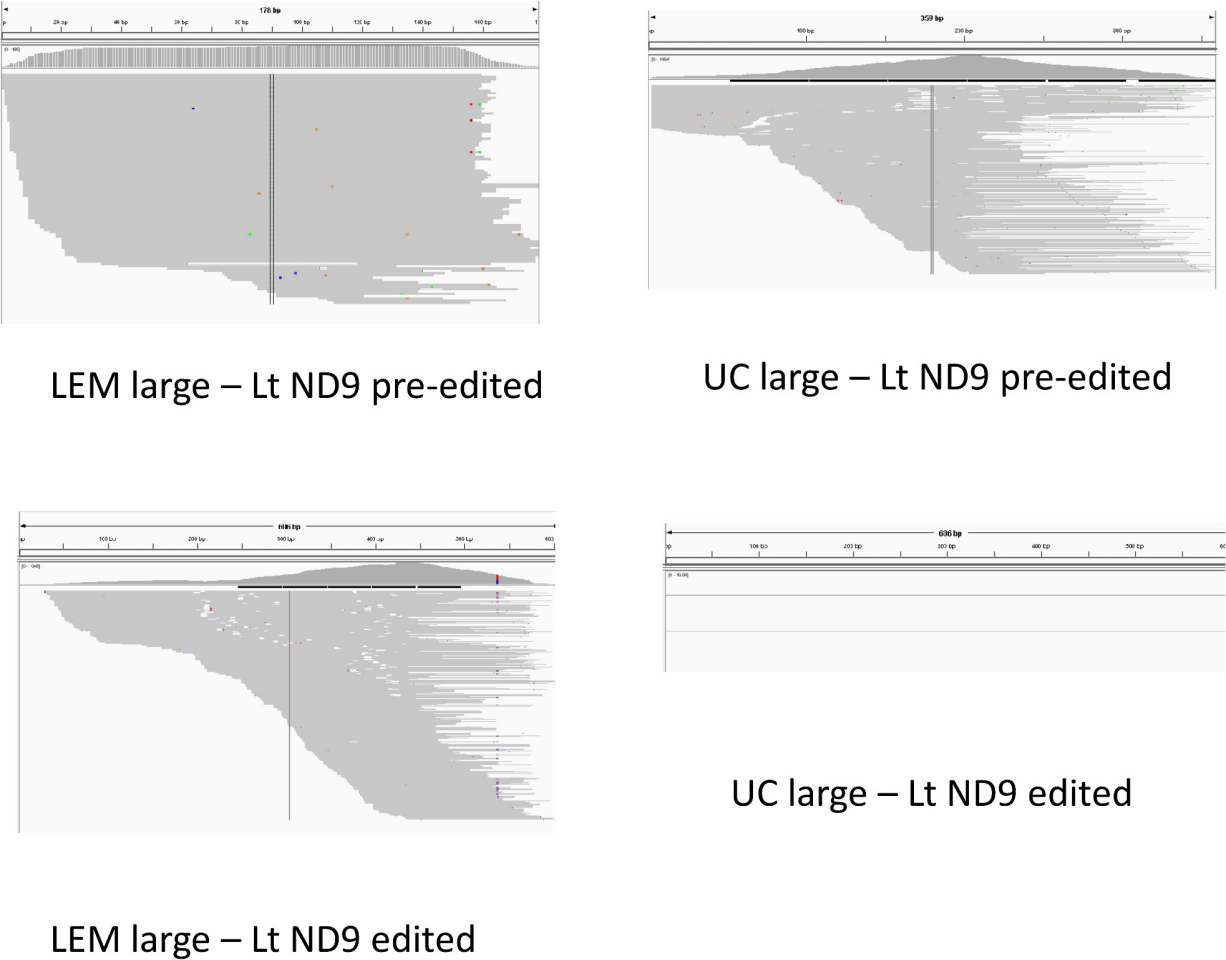
Mapping of large RNA reads to ND9 pre-edited and edited genes. Note the apparent absence of a UC peak for ND9 edited.

**Fig 27:**
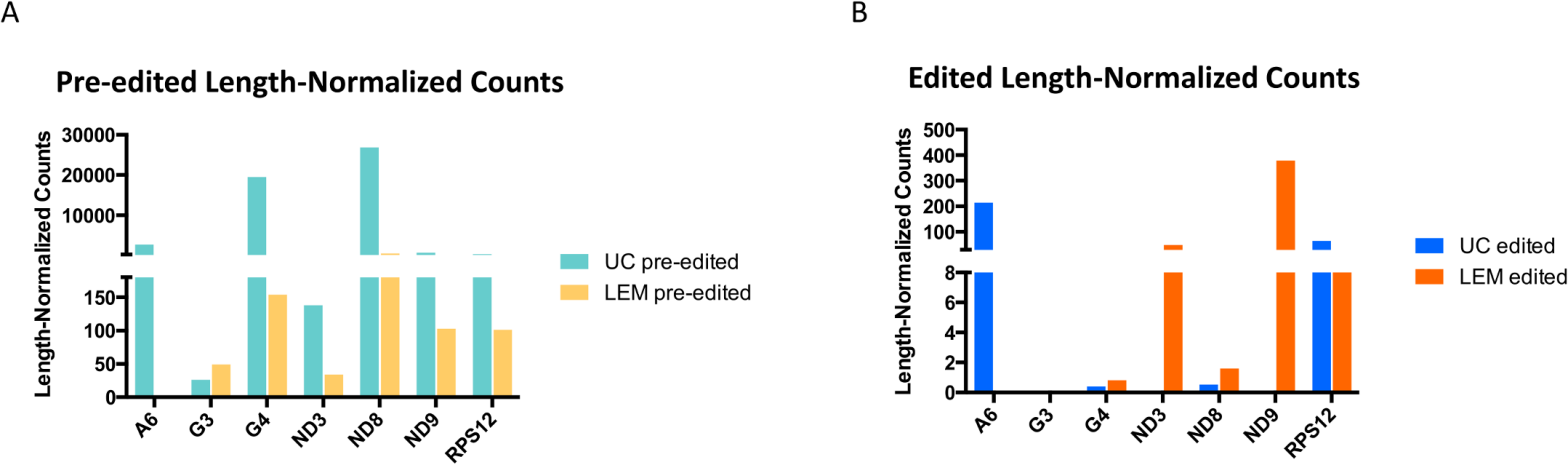
FPKM analysis of the mapping data of pre-edited and mature edited sequences for both strains. A. Pre-edited. B. Edited. The LEM125 strain has substantial edited peaks for ND3, ND9 and RPS12 whereas the UC strain only has substantial edited peaks for A6 and RPS12.

To study strain variations in the extent of editing of the maxicircle gene transcripts, we performed mapping of RNA reads from both strains onto the pre-edited (i.e., genomic) and mature edited maxicircle gene sequences. In most cases the large RNA reads were used, but the small RNA reads gave better visualization for the ND8 and G4 genes. See S20 and S21 Figs for the complete dataset of IGV mapping images. Fig 26 shows a representative mapping experiment using the ND9 pre-edited and edited sequences and large RNA reads from both strains. The absence of ND9 editing in the lower right panel is clear. An FPKM analysis of the mapping data of all pre-edited and mature edited maxicircle genes for both strains is shown in Fig 27. The LEM125 strain shows barely detectable levels of editing for A6, G3, G4 and ND8, which is puzzling since there are complete cascades of minicircle-encoded overlapping gRNAs for A6, and ND8 (Figs 7 and 8). However, since edited sequences were previously obtained by RT PCR for the LEM125 A6, ND8 and G4 genes in our 1994 project [40], lack of detection in the current experiments may be due to a low level of expression and/or an insufficient number of reads in the database for detection. The LEM125 G4 editing cascade, however, apparently lacks two overlapping gRNAs (Fig 9), the loss of which would break the editing cascade. It is of course possible that the database of reads is also insufficient to detect G4 gRNAs, but it is also possible that the specific minicircle classes encoding these two G4 gRNAs were lost in the last 20 years of sporadic culture of LEM125 cells. In the case of the UC strain, however, the absence of editing of ND3, ND8, ND9, G3 and G4 is consistent with the lack of cascades of overlapping minicircle-encoded gRNAs for these genes (Figs 8 and 9).

## Discussion

Due to the presence of only a single gRNA gene per minicircle, the ease of culture and the availability of strains which differ in editing capabilities, the non-pathogenic lizard parasite, *L*. *tarentolae*, provides a model system to investigate this type of RNA editing. The number of catenated minicircles in the kDNA network doubles during S phase and halves in M phase. The network is attached to the mitochondrial membrane and flagellar basal body by a fibrillar apparatus [30, 31]. Minicircles are removed from the network and replicated and recatenated to the periphery at two antipodal sites [64]. The copy numbers of the different minicircle sequence classes in the network fluctuate in nature even during life in culture [37], which has been utilized in "schizodeme analysis" [65] in which gel profiles of restriction enzyme digested kDNA can be used to distinguish strains of trypanosomatid parasites.

The number of 800–1000 nt PacBio CCS sequences derived from single cleaved kinetoplast DNA minicircles of the LEM125 and UC strains which assembled into different minicircle sequences provided a method to quantitate the copy number of different minicircle sequence classes in the network. We confirmed that the number of classes varies dramatically between the UC strain and the LEM125 strain, from around 20–24 for the former to well over 100 for the latter. Multiple alignments of the sequences that assembled into different minicircle sequence classes showed a background of single nucleotide insertions, deletions and substitutions produced in part by sequencing errors, but 61% of the assemblies contained conserved patterns of single nucleotide substitutions. The model is that an original minicircle for a specific gRNA had multiple single nucleotide mutations and that these were preserved after multiple rounds of replication of the molecules. We have calculated that it is very unlikely that these have arisen by chance, suggesting that these changes conferred some type of selective advantage on these cells in culture, the nature of which is unknown.

The gRNA genes which were putatively identified by complementarity with mature edited mRNA sequences, allowing both G/C and G/U base pairs, were shown to be present as steady state transcription products by showing that mitochondrial RNA reads mapped to these genes. The fact that the 5’ ends of the mapped gRNA peaks are frequently flush suggests these are primary transcripts and is consistent with the previously described presence of 5’ triphosphates [2]. However, no bacterial or mitochondrial promoter motifs could be recognized 5’ of the gRNA genes, but the bent DNA region [56, 58, 66–79] which is located approximately 100 bp upstream of the gRNA genes in all minicircles is a possible candidate for transcription initiation since there is a fairly large literature [68, 80–82] on a role for DNA bending in binding transcription or replication factors and in transcription initiation, but this requires further research.

The fact that large RNAs map in some cases to almost the entire minicircle suggests complete transcription of the minicircle. The presence of substantial numbers of non-gRNA reads mapping to both the minus and plus strands suggests bidirectional transcription. Since these peaks contain no identifiable motifs and there is no conservation of the relative localization of these peaks between different minicircles, we suggest that they represent cleavage products of gRNA processing which are relatively more stable to degradation. It is possible however that they have a functional significance, but this remains to be investigated.

The heterogeneity of the 3’ ends of the mapped gRNAs is consistent with 3’ end processing yielding the mature gRNAs. A large proportion of the gRNA transcripts lack varying extents of the 3’ end but these partial gRNAs are probably functional since the 5' anchor sequence is present and the edited region could then be extended by the complete gRNAs.

As mentioned above, there is a moderate level of correlation of transcription with minicircle copy number for the LEM125 strain and somewhat less for the UC strain. For example the LEM125 strain has a single minicircle class encoding a redundant gRNA, gND9VIIb, which represents approximately 20% of the minicircle population and shows a relatively high abundance of steady state transcripts. However, another minicircle representing 6% of the population does not show any enhanced transcript abundance. The remainder of the LEM minicircles vary in abundance from ~1%–2% and show relatively low variable amounts of steady state transcripts. The UC strain has two redundant minicircle classes encoding “orphan” redundant gRNAs for gND3IX representing around 80% of the network which both show high levels of stable gRNA transcripts. This is also the case for a minicircle that represents 6% of the network, but on the other hand two minicircles which represent 6% and 3% of the network show no enhanced level of transcripts. The remainder of the ~24 UC minicircles vary in abundance from 1–5% without any correlation of expression with copy number.

The observed lack of a high correlation of gRNA expression with total minicircle expression suggests that processing and/or turnover may determine the relative abundance of mature gRNAs. And if the minicircle transcript counts for both strains are normalized on a per molecule basis there are fluctuations between steady state expression levels of different minicircles which have no correlation with network copy number. The nature of the regulation of minicircle expression is unknown.

The 114 identified LEM125 gRNAs and the 7 maxicircle-encoded gRNAs form multiple overlapping editing cascades on the pan-edited genes in LEM125. Most of these cascades are functional in editing in this strain and yield mature pan-edited RNAs for RPS12, ND3 and ND9, as determined by mapping RNA reads to mature edited sequences. However, the very low levels of editing of A6, ND8 and G4 in LEM125 in spite of the presence of apparently complete gRNA cascades (Figs 7–9) possibly suggest either an incomplete database of reads or the absence of specific gRNAs which breaks the editing cascade, possibly by loss of the minicircle sequence classes in the 20 years when LEM125 was sporadically cultured. Another possibility is the existence of another level of regulation of expression. In the case of the UC strain the ~24 minicircle-encoded gRNAs only form overlapping cascades with A6 and RPS12, which is consistent with the observed absence of editing for ND3, ND8, ND9, G3 and G4. The lack of editing for G3 in both strains is tentative since there are indications of possible errors in the edited sequence [41].

The lack of editing of several respiratory complex I NADH dehydrogenase subunit genes of the UC strain is reminiscent of the situation in *T*. *brucei* in which insect phase procyclic trypanosomes possibly show developmental down regulation of editing of the NADH dehydrogenase genes. These cells were in fact shown to lack complete gRNA cascades for these genes [42], possibly indicating a loss of minicircle sequence classes. In *Leishmania*, cultured cells exhibit the promastigote form which is characteristic of the midgut stages in the sandfly vector, and, in view of the *T*. *brucei* data, NADH dehydrogenase may not be required for life in culture. This suggests a possible explanation for the observed loss of some minicircle-encoded gRNAs for editing of these genes in culture [37]. It should be noted that both the African trypanosome and *Leishmania* species show several additional developmental changes during the life cycles in the insect and vertebrate hosts and these different stages of the life cycle may exhibit different expression of mitochondrial proteins and in some cases different extents of RNA editing of maxicircle transcripts [83].

We have analyzed the unusual mitochondrial genome of *L*. *tarentolae* in which 12 of the 18 maxicircle-encoded genes contain multiple frameshifts that are normally corrected at the RNA level by U-insertion/deletion RNA editing regulated by base pairing with small guide RNAs which are mainly encoded in the thousands of catenated minicircles. We presented mapping evidence that the minicircles are apparently completely transcribed from both strands with 3' end processing yielding mature gRNAs. The size, location and polarity of the gRNA genes relative to the CSB and "bend" sequences are fairly well conserved. The minicircles form multiple homologous "sequence classes" which each encode a specific gRNA (or redundant gRNA). The number of different sequence classes varies from over 100 in the recently isolated LEM125 strain to only around 20 in the old laboratory UC strain. The frequency of minicircles in different sequence classes is dynamic and can in some cases vary dramatically. A few minicircles apparently do not encode gRNAs. We showed by mapping pre-edited and edited maxicircle sequences that the UC strain lacks editing of five genes, which can be explained as due to the loss of multiple minicircle classes normally encoding gRNAs involved in these editing cascades.

This data suggests that kinetoplastid U-insertion/deletion RNA editing is a dynamic genetic feature. This is probably due to the presence of maxicircle cryptogenes with multiple frameshifts and gRNA genes mainly encoded by minicircles that specify *in trans* by base pairing the information for the precise insertion/deletion of U-residues to overcome the encoded frameshifts.

## Supporting Information

S1 Fig Bar graph of distance from bend to gRNA in 100 minicircles. (TIF)

S2 Fig Bend regions of minicircles and conserved nucleotide changes. A-F. Alignment of bend sequences from several minicircles showing the presence of runs of A and T’s approximately every turn of the helix. Shown graphically in the logo and the diagram of the helix in A.
(TIF)

S3 Fig RNA/mRNA local homology alignments. A-D. The putative minicircle-encoded gRNAs/edited mRNA alignments are shown, identified by the mc number of the minicircle. The duplex minicircle sequence was selected for each alignment. The numbers of the lower edited mRNA sequence refer to the location in a fasta file of the 12 mature edited sequences concatenated head to tail: 1–633 CO2ed, 634–1537 CO3ed, 1538–1867 RPS12ed, 1868–2967 Murf2ed, 2968–3158 G3ed, 3159–3695 G4ed, 3696–4845 Cybed, 4846–5040 A6ed, 5041–5449 ND3ed, 5450–6649 ND7ed, 6650–7169 ND8ed, 7170–7821 ND9ed.
(TIF)

S4 Fig RNA/mRNA local homology alignments. A-D. See S3 legend for details.
(TIF)

S5 Fig RNA/mRNA local homology alignments. A-D. See S3 legend for details.
(TIF)

S6 Fig RNA/mRNA local homology alignments. A-D. See S3 legend for details.
(TIF)

S7 Fig RNA/mRNA local homology alignments. A-B. See S3 legend for details.
(TIF)

S8 Fig Mapping of reads from RNA libraries from both strains against 114 minicircles. The small RNA library represents merging of Bam files from alignments of reads in libraries derived from small RNAs isolated from isolated kinetoplast fractions and small RNAs from total cell RNA. The large RNA library was constructed from RNA isolated from kinetoplast fractions and represents merging of Bam files from alignments of unpaired reads. The specific minicircles and the computer-identified gRNA genes are annotated below the maps. In A the peaks of reads that map to the gRNA genes are circled. In B and C, the more extensive mappings mainly of the large RNA reads are boxed.
(TIF)

S9 Fig Mapping of reads from RNA libraries from both strains against 114 minicircles. See S8 legend for details. A-D. The more extensive mappings mainly of the large RNA reads are boxed.
(TIF)

S10 Fig Mapping of reads from RNA libraries from both strains against 114 minicircles. See S8 legend for details. A-D. The more extensive mappings mainly of the large RNA reads are boxed.
(TIF)

S11 Fig Mapping of reads from RNA libraries from both strains against 114 minicircles. See S8 legend for details. A-B. The more extensive mappings mainly of the large RNA reads are boxed. C. Several of the minicircles with no identified gRNA have gRNA-like mapped peaks which are indicated in red.
(TIF)

S12 Fig Representative nucleotide profiles of mapped gRNA reads using the small RNA library. A-D. The annotated gRNA box at the bottom is linked by a rectangular box to the mapped peak of reads. The actual nucleotide reads in the gRNA peak are shown on the right. At the top is the total number of reads in the peak.
(TIF)

S13 Fig Representative nucleotide profiles of mapped gRNA reads using the small RNA library. A-D. The annotated gRNA box at the bottom is linked by a rectangular box to the mapped peak of reads. The actual nucleotide reads in the gRNA peak are shown on the right. At the top is the total number of reads in the peak.
(TIF)

S14 Fig Representative nucleotide profiles of mapped gRNA reads using the small RNA library. A-D. The annotated gRNA box at the bottom is linked by a rectangular box to the mapped peak of reads. The actual nucleotide reads in the gRNA peak are shown on the right. At the top is the total number of reads in the peak.
(TIF)

S15 Fig Representative nucleotide profiles of mapped gRNA reads using the small RNA library. A-D. The annotated gRNA box at the bottom is linked by a rectangular box to the mapped peak of reads. The actual nucleotide reads in the gRNA peak are shown on the right. At the top is the total number of reads in the peak.
(TIF)

S16 Fig Representative nucleotide profiles of mapped gRNA reads using the small RNA library. The annotated gRNA box at the bottom is linked by a rectangular box to the mapped peak of reads. The actual nucleotide reads in the gRNA peak are shown on the right. At the top is the total number of reads in the peak.
(TIF)

S17 Fig Mapping of UC and LEM short RNA reads to the 100 minicircle-encoded gRNAs. A-B. Upper panel—UC small RNA merged Bam files for small mitochondrial + small total cell RNA. Lower panel–LEM125 small RNA merged Bam files. C-D. Same dataset as in A-B but at a higher magnification. Reads from the plus strand are red and those from the minus strand are blue. The gRNAs are annotated as blue boxes and labeled.
(TIF)

S18 Fig Mapping of UC and LEM short RNA reads to the 100 minicircle-encoded gRNAs. A-D. Reads from the plus strand are red and those from the minus strand are blue. The gRNAs are annotated as blue boxes and labeled.
(TIF)

S19 Fig Mapping of UC and LEM short RNA reads to the 100 minicircle-encoded gRNAs. A-D. Reads from the plus strand are red and those from the minus strand are blue. The gRNAs are annotated as blue boxes and labeled.
(TIF)

S20 Fig Mapping RNA reads to pre-edited and edited sequences. The edited sequences mapped do not include any pre-edited sequence. The reads are usually “squished” for visualization in IGV. A. Large RNA reads from both strains mapped to A6 pre-edited and edited sequences. B. Large RNA reads from both strains mapped to pre-edited and edited RPS12 sequences. C. Large RNA reads from both strains mapped to pre-edited and edited ND3 sequences. Note absence of mapped edited ND3 reads for UC strain. D. Small RNA reads from both strains mapped to pre-edited and edited ND8 sequences. Note the absence of mapped edited ND8 reads for both strains.
(TIF)

S21 Fig Mapping RNA reads to pre-edited and edited sequences. A. Large RNA reads from both strains mapped to pre-edited and edited G3 sequences. Note absence of mapped edited G3 reads for both strains. B. Small RNA reads from both strains mapped to pre-edited and edited G4 sequences. Note absence of mapped G4 sequences for both strains.
(TIF)
