# Supplementary figures and images for: Comparison of the Mitochondrial Genomes and Steady State Transcriptomes of Two Strains of the Trypanosomatid Parasite, Leishmania tarentolae
Source: PLoS Negl Trop Dis. 2015 Jul 23;9(7):e0003841. doi: 10.1371/journal.pntd.0003841 (PMC4512693; doi:10.1371/journal.pntd.0003841)

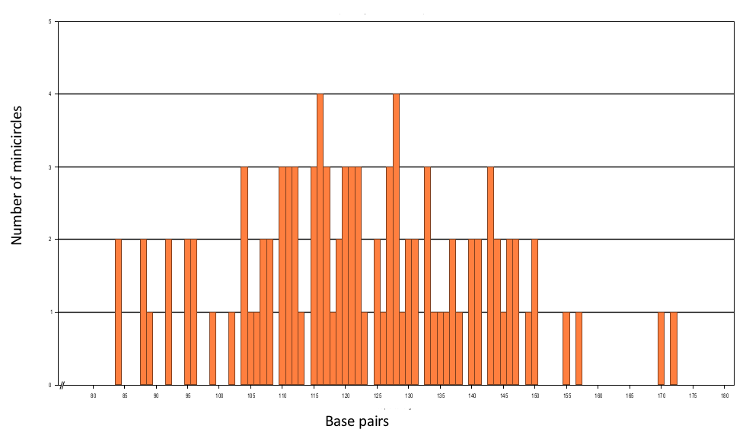

Supplement: S1 Fig — (TIF) [file pntd.0003841.s001.tif]

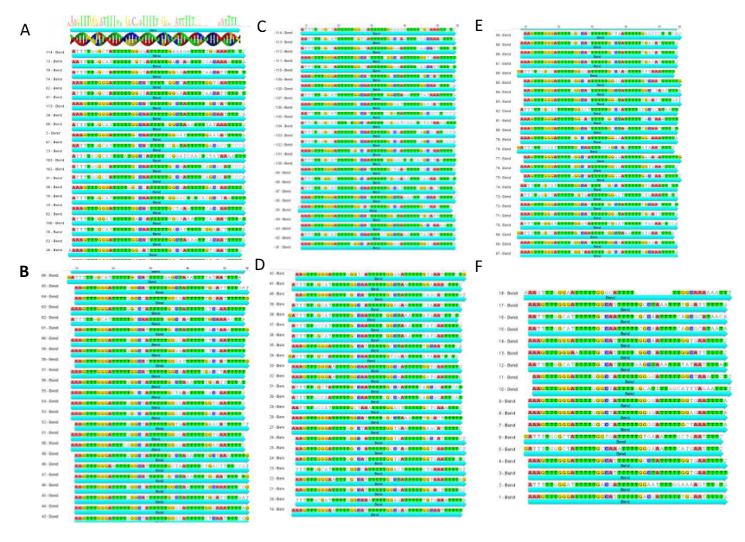

Supplement: S2 Fig — A-F. Alignment of bend sequences from several minicircles showing the presence of runs of A and T’s approximately every turn of the helix. Shown graphically in the logo and the diagram of the helix in A. (TIF) [file pntd.0003841.s002.tif]

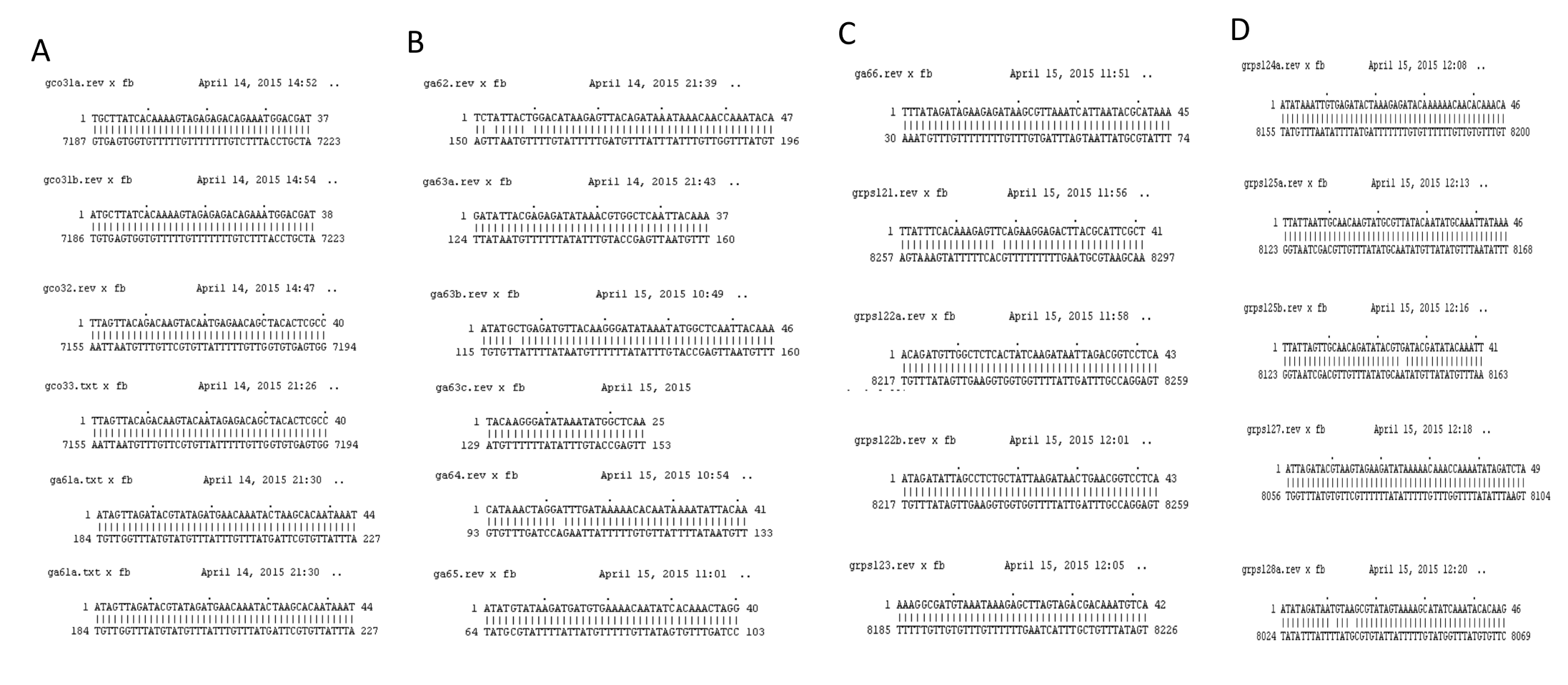

Supplement: S3 Fig — A-D. The putative minicircle-encoded gRNAs/edited mRNA alignments are shown, identified by the mc number of the minicircle. The duplex minicircle sequence was selected for each alignment. The numbers of the lower edited mRNA sequence refer to the location in a fasta file of the 12 mature edited sequences concatenated head to tail: 1–633 CO2ed, 634–1537 CO3ed, 1538–1867 RPS12ed, 1868–2967 Murf2ed, 2968–3158 G3ed, 3159–3695 G4ed, 3696–4845 Cybed, 4846–5040 A6ed, 5041–5449 ND3ed, 5450–6649 ND7ed, 6650–7169 ND8ed, 7170–7821 ND9ed. (TIF) [file pntd.0003841.s003.tif]

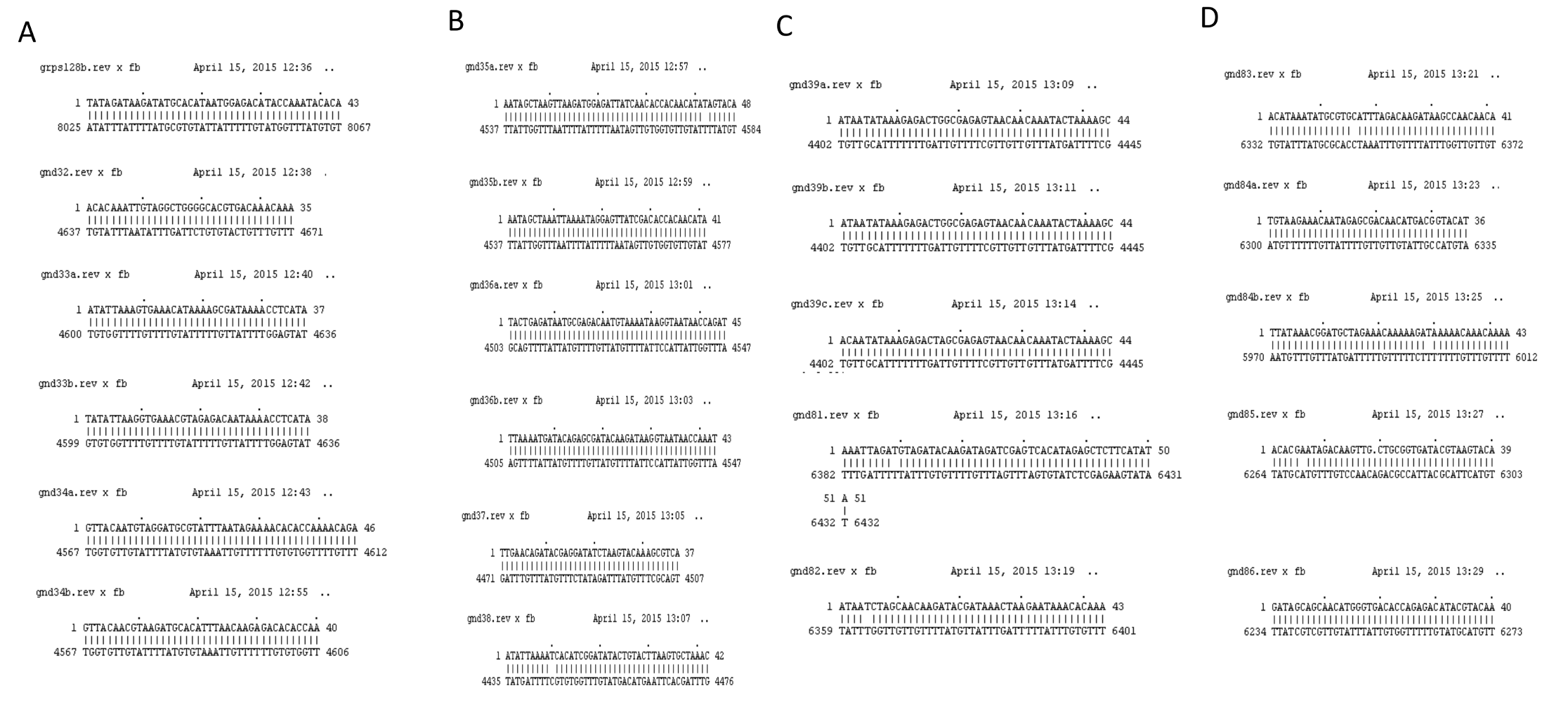

Supplement: S4 Fig — A-D. See S3 legend for details. (TIF) [file pntd.0003841.s004.tif]

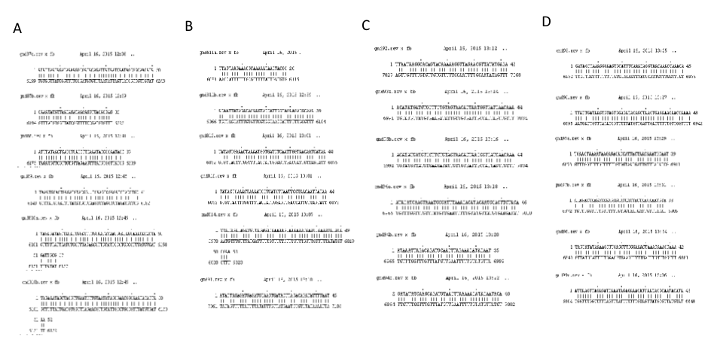

Supplement: S5 Fig — A-D. See S3 legend for details. (TIF) [file pntd.0003841.s005.tif]

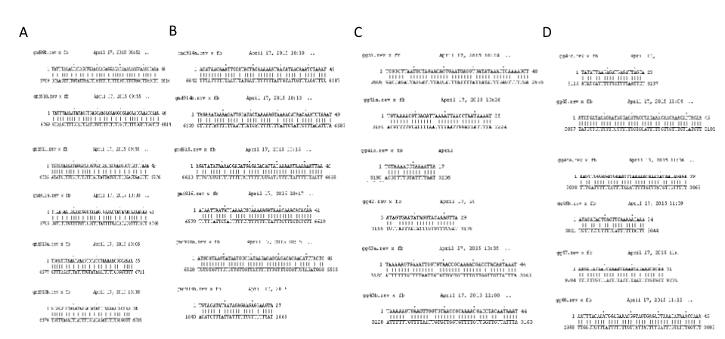

Supplement: S6 Fig — A-D. See S3 legend for details. (TIF) [file pntd.0003841.s006.tif]

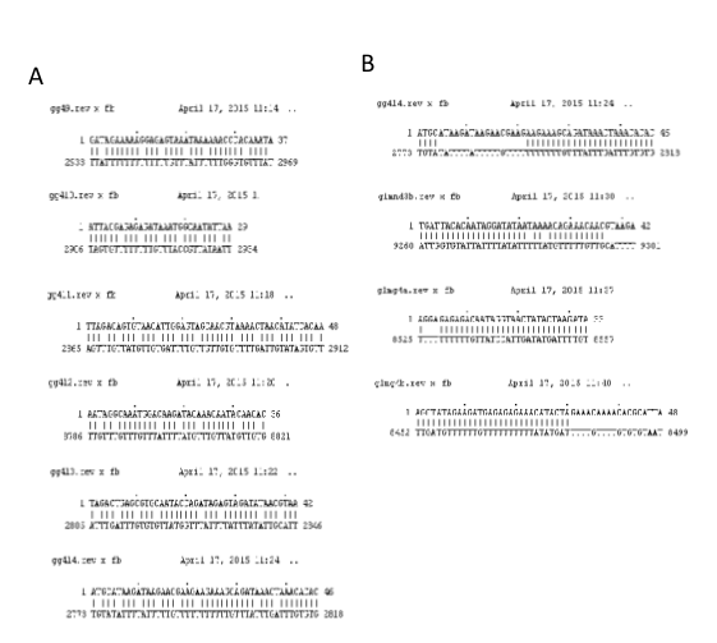

Supplement: S7 Fig — A-B. See S3 legend for details. (TIF) [file pntd.0003841.s007.tif]

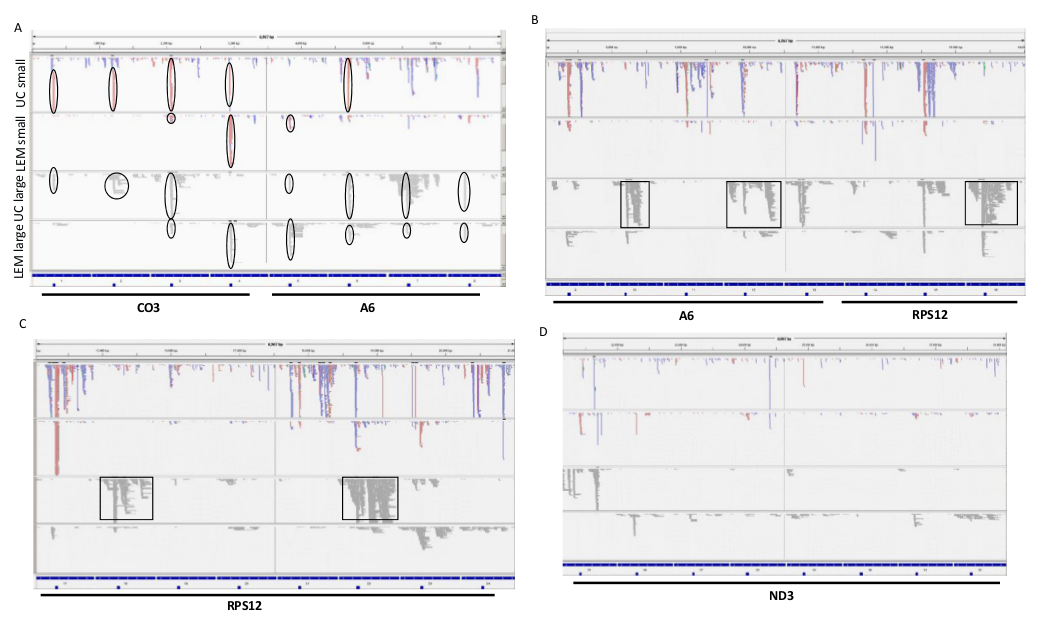

Supplement: S8 Fig — The small RNA library represents merging of Bam files from alignments of reads in libraries derived from small RNAs isolated from isolated kinetoplast fractions and small RNAs from total cell RNA. The large RNA library was constructed from RNA isolated from kinetoplast fractions and represents merging of Bam files from alignments of unpaired reads. The specific minicircles and the computer-identified gRNA genes are annotated below the maps. In A the peaks of reads that map to the gRNA genes are circled. In B and C, the more extensive mappings mainly of the large RNA reads are boxed. (TIF) [file pntd.0003841.s008.tif]

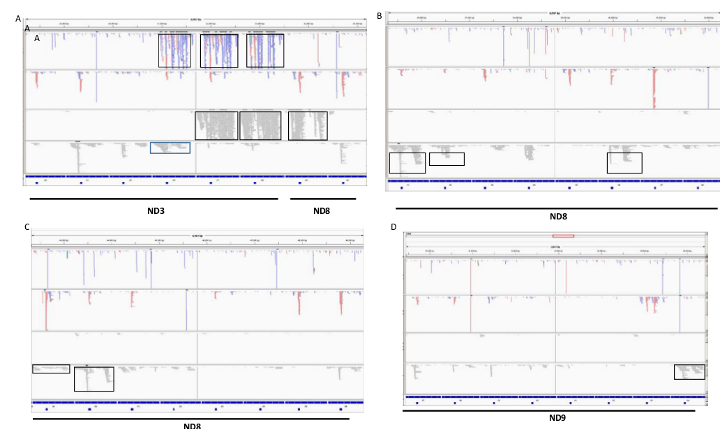

Supplement: S9 Fig — See S8 legend for details. A-D. The more extensive mappings mainly of the large RNA reads are boxed. (TIF) [file pntd.0003841.s009.tif]

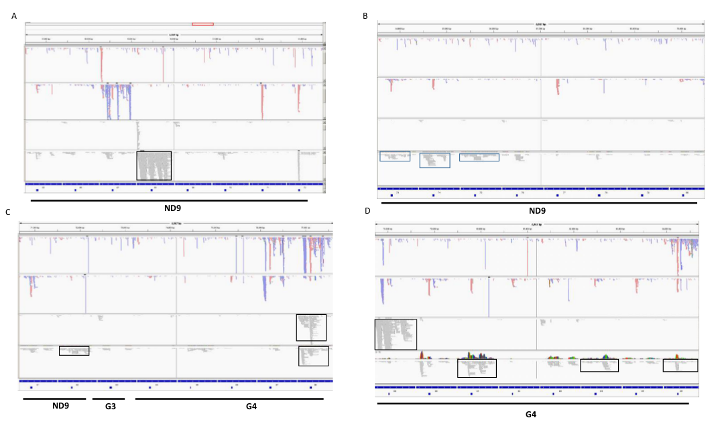

Supplement: S10 Fig — See S8 legend for details. A-D. The more extensive mappings mainly of the large RNA reads are boxed. (TIF) [file pntd.0003841.s010.tif]

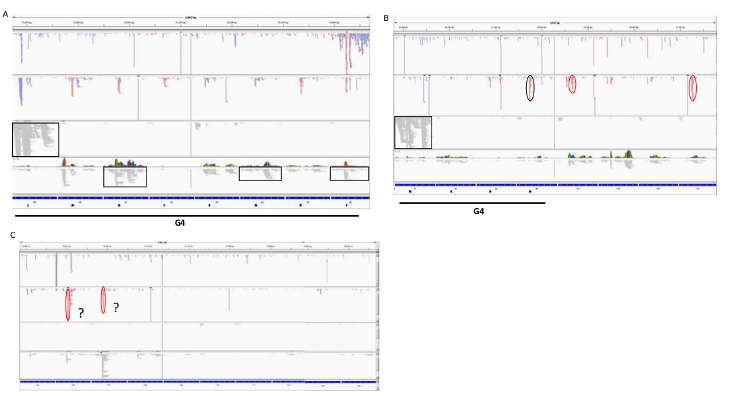

Supplement: S11 Fig — See S8 legend for details. A-B. The more extensive mappings mainly of the large RNA reads are boxed. C. Several of the minicircles with no identified gRNA have gRNA-like mapped peaks which are indicated in red. (TIF) [file pntd.0003841.s011.tif]

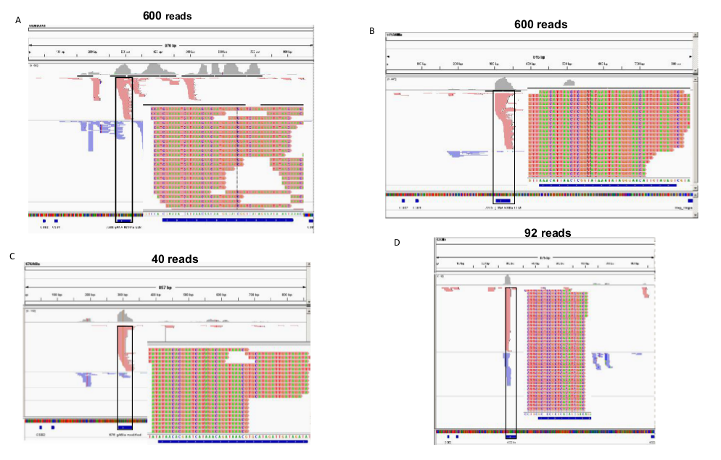

Supplement: S12 Fig — A-D. The annotated gRNA box at the bottom is linked by a rectangular box to the mapped peak of reads. The actual nucleotide reads in the gRNA peak are shown on the right. At the top is the total number of reads in the peak. (TIF) [file pntd.0003841.s012.tif]

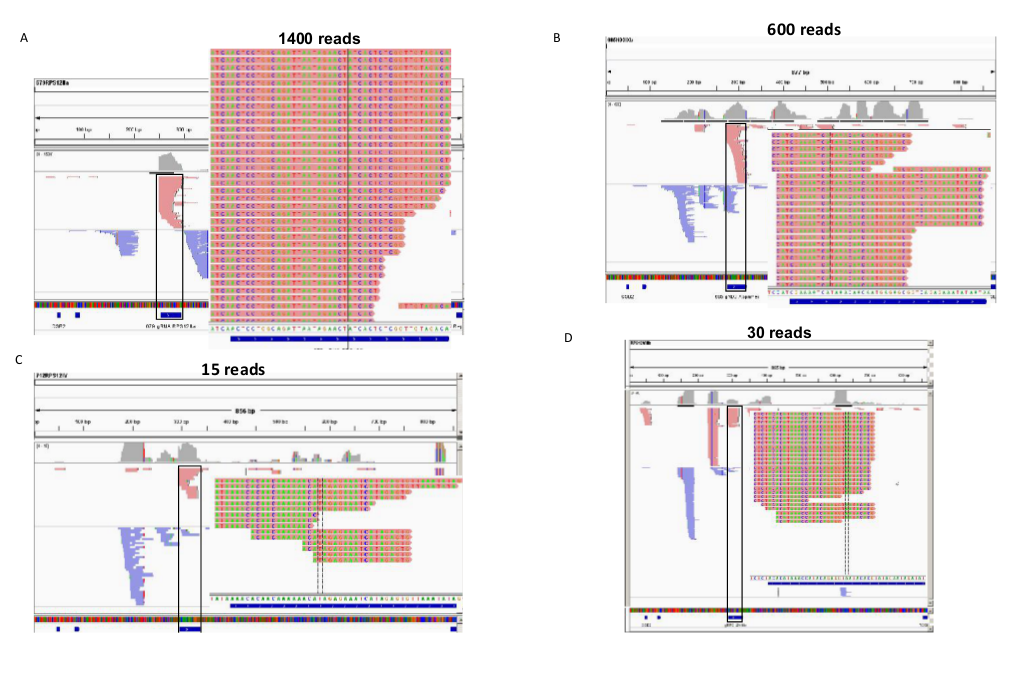

Supplement: S13 Fig — A-D. The annotated gRNA box at the bottom is linked by a rectangular box to the mapped peak of reads. The actual nucleotide reads in the gRNA peak are shown on the right. At the top is the total number of reads in the peak. (TIF) [file pntd.0003841.s013.tif]

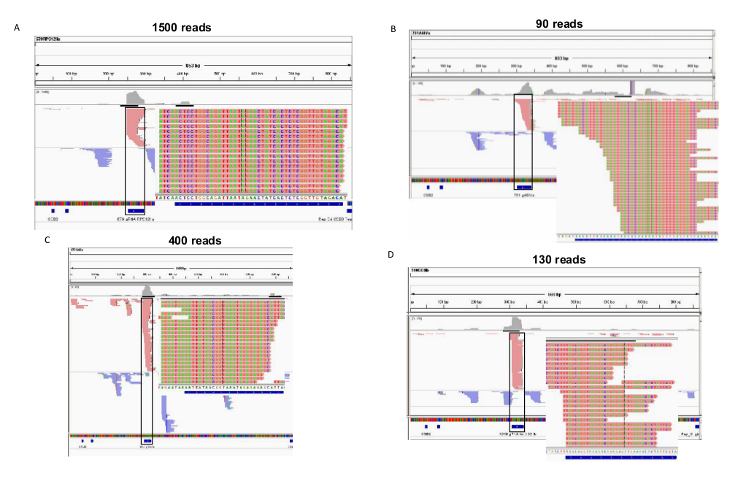

Supplement: S14 Fig — A-D. The annotated gRNA box at the bottom is linked by a rectangular box to the mapped peak of reads. The actual nucleotide reads in the gRNA peak are shown on the right. At the top is the total number of reads in the peak. (TIF) [file pntd.0003841.s014.tif]

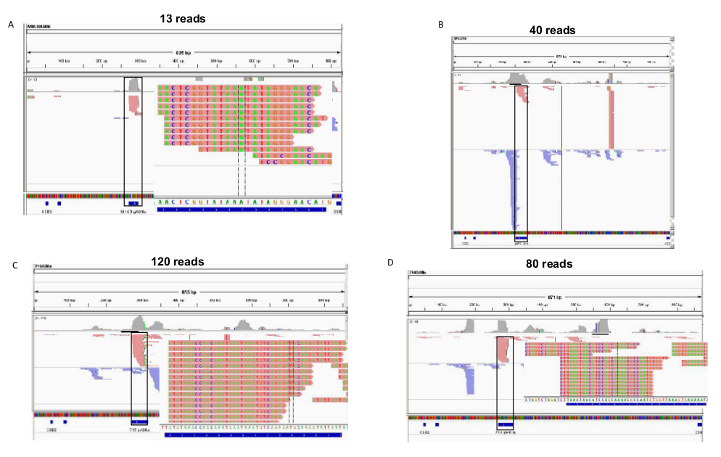

Supplement: S15 Fig — A-D. The annotated gRNA box at the bottom is linked by a rectangular box to the mapped peak of reads. The actual nucleotide reads in the gRNA peak are shown on the right. At the top is the total number of reads in the peak. (TIF) [file pntd.0003841.s015.tif]

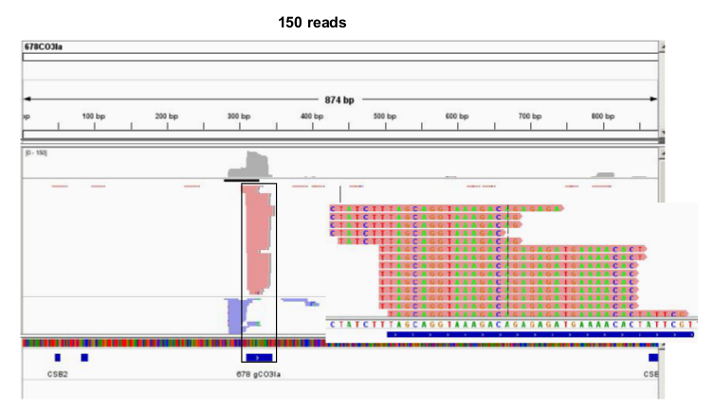

Supplement: S16 Fig — The annotated gRNA box at the bottom is linked by a rectangular box to the mapped peak of reads. The actual nucleotide reads in the gRNA peak are shown on the right. At the top is the total number of reads in the peak. (TIF) [file pntd.0003841.s016.tif]

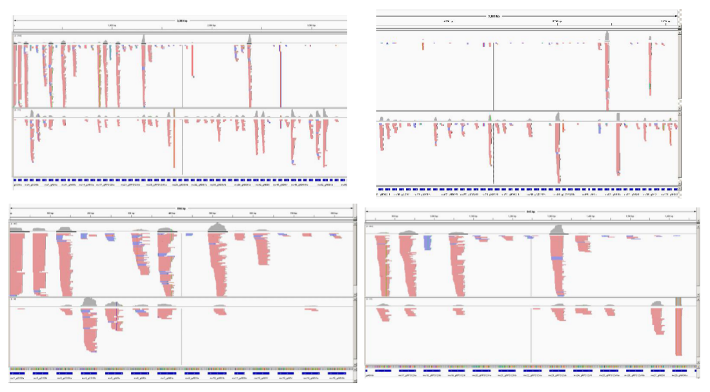

Supplement: S17 Fig — A-B. Upper panel—UC small RNA merged Bam files for small mitochondrial + small total cell RNA. Lower panel–LEM125 small RNA merged Bam files. C-D. Same dataset as in A-B but at a higher magnification. Reads from the plus strand are red and those from the minus strand are blue. The gRNAs are annotated as blue boxes and labeled. (TIF) [file pntd.0003841.s017.tif]

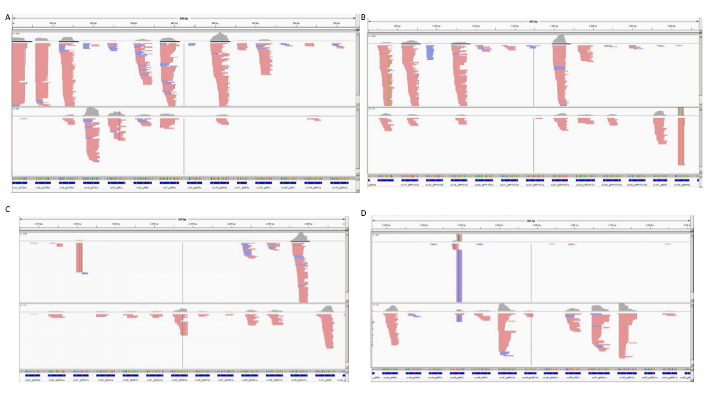

Supplement: S18 Fig — A-D. Reads from the plus strand are red and those from the minus strand are blue. The gRNAs are annotated as blue boxes and labeled. (TIF) [file pntd.0003841.s018.tif]

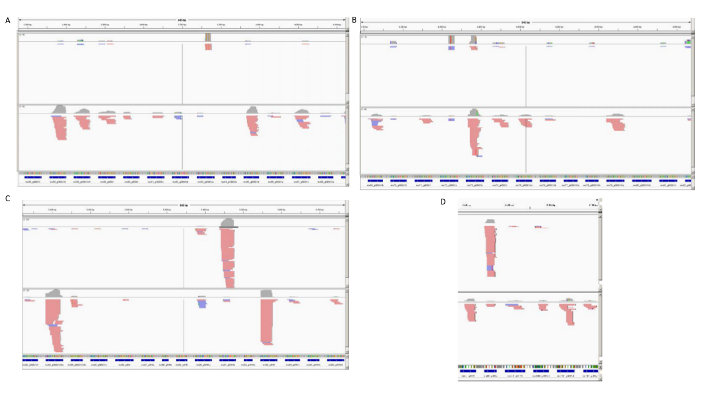

Supplement: S19 Fig — A-D. Reads from the plus strand are red and those from the minus strand are blue. The gRNAs are annotated as blue boxes and labeled. (TIF) [file pntd.0003841.s019.tif]

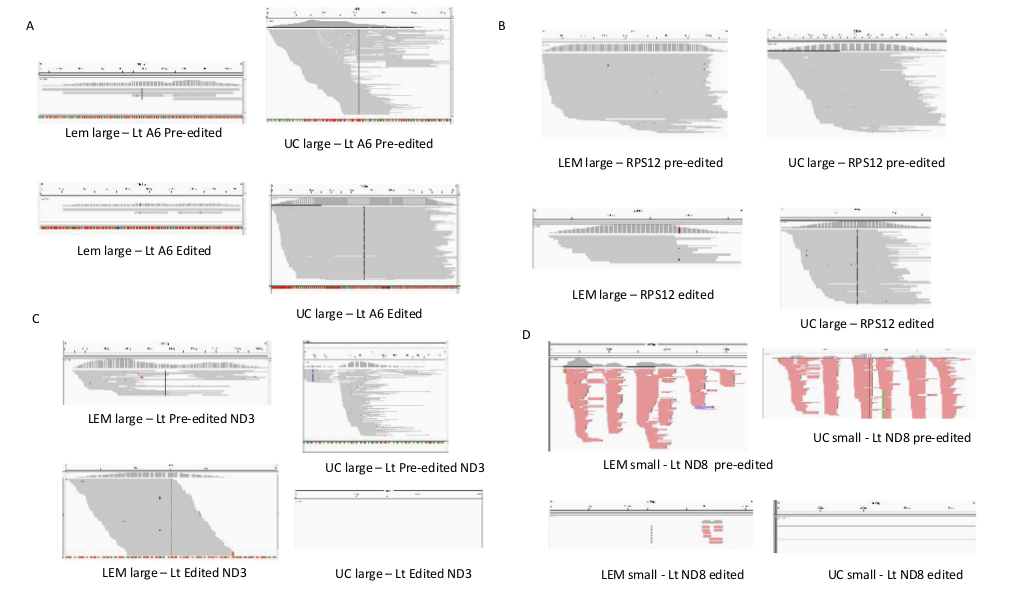

Supplement: S20 Fig — The edited sequences mapped do not include any pre-edited sequence. The reads are usually “squished” for visualization in IGV. A. Large RNA reads from both strains mapped to A6 pre-edited and edited sequences. B. Large RNA reads from both strains mapped to pre-edited and edited RPS12 sequences. C. Large RNA reads from both strains mapped to pre-edited and edited ND3 sequences. Note absence of mapped edited ND3 reads for UC strain. D. Small RNA reads from both strains mapped to pre-edited and edited ND8 sequences. Note the absence of mapped edited ND8 reads for both strains. (TIF) [file pntd.0003841.s020.tif]

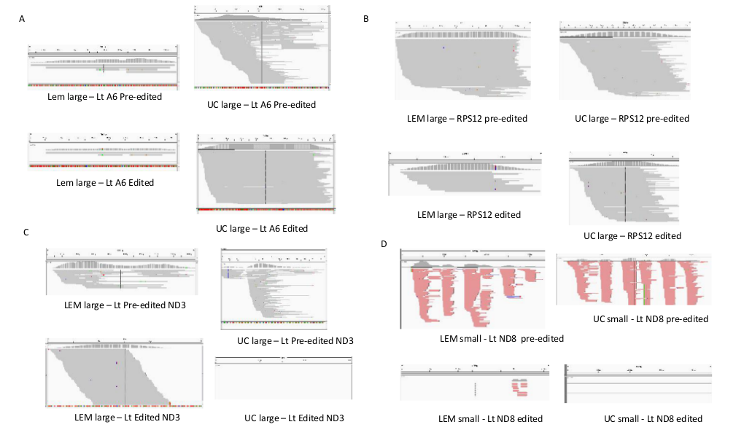

Supplement: S21 Fig — A. Large RNA reads from both strains mapped to pre-edited and edited G3 sequences. Note absence of mapped edited G3 reads for both strains. B. Small RNA reads from both strains mapped to pre-edited and edited G4 sequences. Note absence of mapped G4 sequences for both strains. (TIF) [file pntd.0003841.s021.tif]
